# Cancer risk from low-dose ionizing radiation in dental imaging: A systematic review and meta-analysis

**DOI:** 10.1186/s12903-026-08522-0

**Published:** 2026-05-04

**Authors:** Kawtar Chadli, Houda Ousli, Houda Neani, Fatima Zaoui, Amal Bouziane, Hicham Benyahia

**Affiliations:** 1 Dentofacial Orthopedics Department, Faculty of Dental Medicine, Mohammed V University in Rabat, BP: 6212, Rabat, Morocco; 2 Department of Periodontology, Faculty of Dental Medicine, Mohammed V University in Rabat, Rabat, Morocco; 3 Laboratory of Biostatistics, Clinical Research and Epidemiology, Mohammed V University in Rabat, Rabat, Morocco

**Keywords:** Dental radiography, Radiation exposure, Head and neck cancer, Meningioma, Thyroid neoplasm, Meta-analysis, Systematic review

## Abstract

**Objective:**

To evaluate the association between low-dose ionizing radiation from dental imaging (conventional radiography and cone-beam/medical CT) and cancer risk using contemporary epidemiological evidence.

**Materials and methods:**

This systematic review and meta-analysis followed PRISMA 2020 guidelines. PubMed, Scopus, and Web of Science were searched up to March 2026 for observational cohort and case-control studies assessing cancer risk after dental radiographic exposure. Risk of bias was assessed using Joanna Briggs Institute tools, causal inference with COSMOS-E guidance, and certainty of evidence with the GRADE framework. Random-effects models pooled adjusted Odds Ratios (ORs) and adjusted Hazard Ratios (HRs) with 95% confidence intervals (CIs).

**Results:**

Among 1,883 records, 24 studies met inclusion criteria, and 19 were included in meta-analysis (415,887 participants). In the 16 case-control studies, thyroid cancer was significantly associated with dental imaging (OR = 2.21; 95% CI: 1.63–2.99), while central nervous system (CNS) tumors showed a non-significant elevation (OR = 1.31; 95% CI: 0.89–1.91). In the three cohort studies, thyroid cancer showed a small but significant association with conventional radiography (HR = 1.13; 95% CI: 1.01–1.26), and CNS tumors risk was moderately associated with CT scan exposure (HR = 1.54; 95% CI: 1.03–2.29). Certainty of evidence was rated low for thyroid cancer, and very low for central nervous system cancers, lymphoid cancer, oral cancer and salivary gland cancer due to risk of bias, inconsistency, and imprecision.

**Conclusion:**

Current evidence is insufficient to confirm an association between low-dose dental radiographic exposure and cancer. A small increased risk was observed, but certainty remains very low, highlighting the need for well-designed prospective research.

**Supplementary Information:**

The online version contains supplementary material available at 10.1186/s12903-026-08522-0.

## Introduction

Recent advances in radiobiology and epidemiology have increased our understanding of the risks associated with low-dose radiation. Medical exposure remains the largest artificial source of radiation worldwide, with approximately 4.2 billion radiological examinations performed annually. Dental radiology accounts for 26.3% of all procedures but contributes only 0.2% of the collective effective dose, whereas computed tomography (CT) scans, which represent 9.6% of procedures, account for 61.6% of the dose burden [1].

Commonly used radiographs in dentistry include periapical, bitewing, panoramic, and lateral cephalograms. There is a growing shift toward three-dimensional radiography, particularly in interceptive orthopedics, orthodontic planning, endodontics, and prosthodontics. As advanced imaging becomes more accessible, both the frequency and variety of radiographs have increased. Although these advances offer greater diagnostic precision, they raise concerns about cumulative radiation exposure and emphasize the need for individualized justification of their use [2].

The primary concern with ionizing radiation is its stochastic risk of cancer induction, particularly during childhood exposure. This is due to rapid cell division and undifferentiated cells during growth, which increase susceptibility to chromosomal damage. A smaller cranial volume exposes sensitive organs such as the thyroid and brain to greater radiation than adults [3]. Additionally, longer life expectancy suggests more exposure, thus higher cumulative doses and a prolonged period for radiation-induced effects to develop.

Cancer risk from low-dose exposure is modeled via the linear no-threshold (LNT) hypothesis, which postulates a proportional relationship between dose and risk without a safe threshold [4]. This model is based on epidemiological data from high-dose exposures, such as Hiroshima, to estimate risks at lower doses [5]. According to the latest report from the IRSN (Institute for Radiation Protection and Nuclear Safety), scientific evidence supports the validity of the LNT model, indicating that even minimal radiation exposure may increase cancer risk [4–6].

In this context, a systematic review of the available epidemiological evidence was conducted to evaluate cancer risks associated with low-dose ionizing radiation (LDIR) from dental imaging. The analysis focuses on large cohort studies of individuals exposed to diagnostic dental X-rays. Cone-beam computed tomography (CBCT) and CT scans were included because of their increasing use in dental and maxillofacial imaging, particularly for orthognathic surgical planning, pediatric trauma management, and complex craniofacial conditions (such as syndromes and cleft anomalies). Although CT scans delivers higher radiation doses than conventional dental X-rays, exposure levels remain within the low-dose range, justifying their inclusion in this assessment.

## Materials and methods

### Protocol and registration

This systematic review was conducted following the Preferred Reporting Items of Systematic Reviews and Meta-Analysis (PRISMA) guidelines [7]. The protocol was registered with the international prospective register of systematic reviews (PROSPERO) under protocol registration number CRD42024628319.

### Eligibility criteria

The systematic review process used the PECO framework (Patient, Exposition, Comparator, Outcomes), which is recognized as an effective tool for conducting systematic reviews. In this review, P represented adults and children, E corresponded to exposure to diagnostic low-dose ionizing radiation in dental settings (conventional radiographs and CT scans), C denoted the absence of dental X-ray exposure, and O encompassed outcomes such as brain cancer, meningioma, thyroid cancer, leukemia, and other malignancies.

Eligible studies met the following criteria: (i) human studies and (ii) investigation of cancers associated with dental radiographic exposure. Observational studies, specifically cohort and case‒control studies, that examined cancer development in patients exposed to diagnostic dental X-rays were considered without language restriction.

Studies were excluded if they involved animal or in vitro designs, case reports, conference abstracts, letters, or focused on occupational radiation exposure (including medical and oral and maxillofacial radiology personnel), or evaluated outcomes other than cancer. Additionally, studies were excluded if they did not report risk estimates adjusted for relevant confounding variables.

### Search strategy

This systematic review searched three databases (PubMed, Scopus, and Web of Science) up to March 2026 and was conducted independently by two reviewers (C.K. and O.H.). The full search strings for all databases are provided in Supplementary File 1. Reference lists of included studies were hand-searched to identify additional relevant articles.

### Study selection

Two reviewers (C.K. and O.H.) independently screened titles and abstracts, followed by full-text assessments based on the eligibility criteria. Any disagreements that occurred were resolved through discussion in consultation with a third reviewer (B.H.) until consensus was reached.

### Data extraction and analysis

Data extraction was independently performed by two reviewers (C.K. and O.H.) via a standardized form. The primary outcome measures were the Odds Ratio (OR) and Relative Risk (RR) for case-control studies, and the Hazard Ratio (HR) and Incidence Rate Ratio (IRR) for cohort studies, with 95% confidence intervals (CIs). The extracted data included study design, sample size, participant demographics, follow-up duration for cohort studies, exposure type, effect estimates and interpretation with 95% CIs and adjustment for confounders.

The risk of bias (RoB) was assessed via the Joanna Briggs Institute (JBI) Critical Appraisal Tools for case‒control and cohort studies [8, 9]. Each item was scored as yes, no, unclear, or not applicable. Studies with higher scores were deemed to have low bias. The results were visualized in a traffic-light plot [10]. In addition, the COSMOS-E guidelines were applied to support the evaluation of causal inference [11]. Key considerations included confounding control, selection bias, information bias, and study sensitivity. Studies were graded as strong, moderate, or weak based on these domains.

### Meta-analysis

A random-effects model was applied to account for anticipated heterogeneity across studies. Given differences in effect measures, separate meta-analyses were conducted for case-control and cohort studies. For case-control studies, adjusted Odds Ratios (ORs) and Relative Risks (RRs) were pooled under the rare disease assumption. The cancers included in this review have a prevalence below 10%, satisfying the criteria for this approximation. For cohort studies, adjusted Hazard Ratios (HRs) were pooled. Incidence Rate Ratios (IRRs) were not pooled due to incompatible data. All pooled estimates are presented with 95% confidence intervals (CIs).

Heterogeneity was assessed via the *I²* statistic, with values > 50% indicating substantial variation. Subgroup analyses were planned based on cancer type, risk of bias, and exposure ascertainment method. Cancers were grouped anatomically into thyroid, central nervous system (including glioma, meningioma, and vestibular schwannoma), lymphoid cancer, oral cancer, and salivary gland cancer to improve interpretability. Sensitivity analyses were performed by omitting studies one at a time for each meta-analysis. Publication bias was assessed via Begg’s and Egger’s tests, along with visual inspection of funnel plots. The analyses were performed with Stata 17.0 (Metan package) [12].

### Certainty of evidence

The overall certainty of evidence was assessed via the GRADE (Grading of Recommendations Assessment, Development and Evaluation) framework, which was applied to each outcome included in the meta-analysis [13]. The observational studies began as low-certainty evidence and were downgraded for (1) serious risk of bias (assessed using JBI and COSMOS-E tools), (2) inconsistency (I² > 50%), (3) indirectness, (4) imprecision (95% confidence intervals crossing null), and (5) potential publication bias. Upgrading was considered in cases of observed dose‒response trends (across ≥ 3 studies), large magnitude of effect (OR/RR > 2), or when plausible confounding would reduce the observed effects. Final certainty ratings were expressed using standard GRADE symbols (High ⊕⊕⊕⊕ to Very Low ⊕⊖⊖⊖) and categorized according to their importance for clinical decision-making.

## Results

### Study selection

The initial database search retrieved 1883 records. After 506 duplicates were removed, 1377 unique records remained. Following title and abstract screening, 1299 articles were excluded because of their irrelevance to the research question, and one article couldn’t be retrieved.

**Fig. 1:**
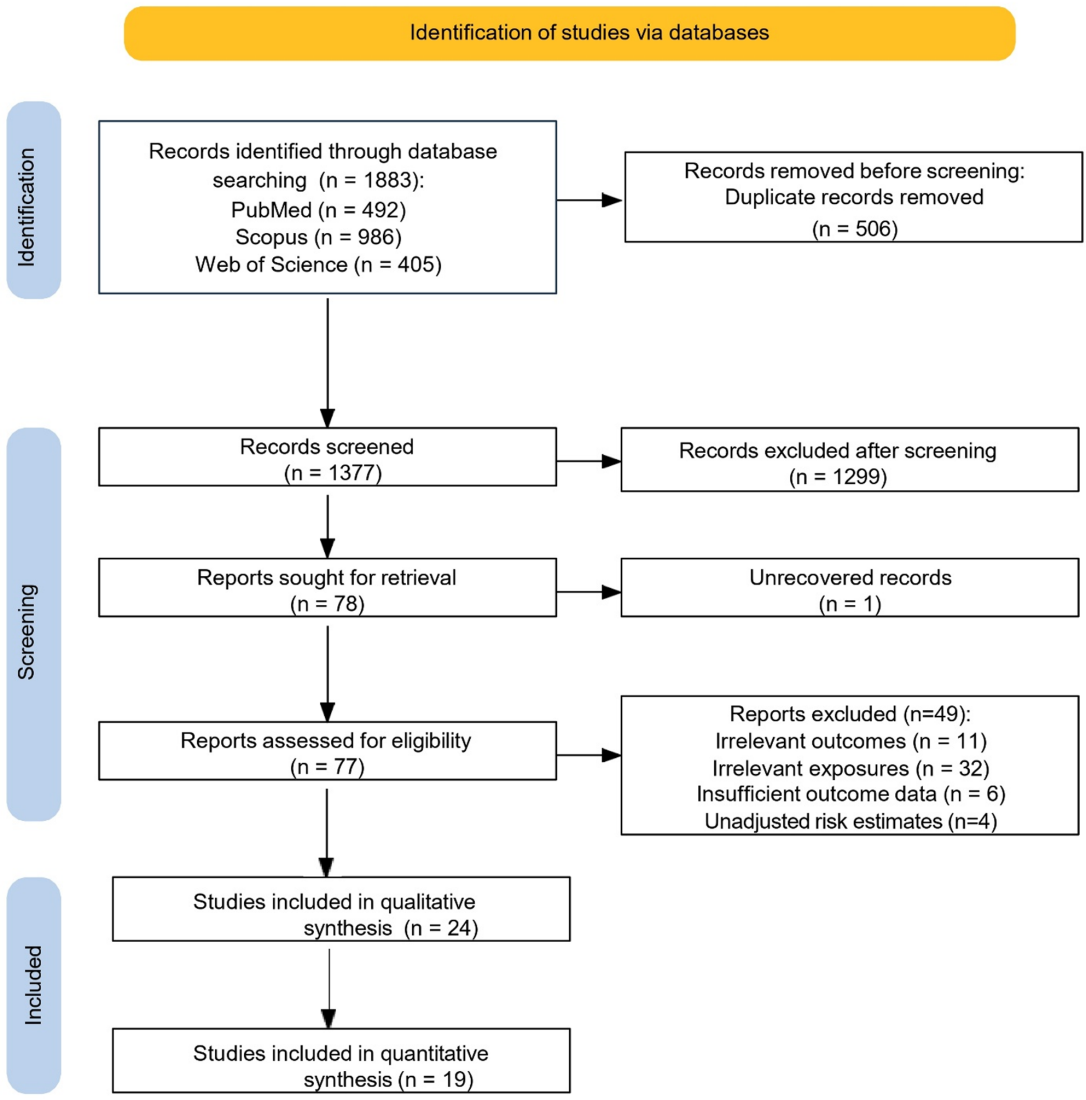
PRISMA 2020 flow diagram showing the selection process of articles for the systematic literature review and meta-analysis

Seventy-seven articles were assessed for eligibility. Among them, 49 were excluded for the following reasons: irrelevant outcomes (*n* = 11), exposure unrelated to dental imaging (*n* = 32), and insufficient outcome data (*n* = 6), and unadjusted risk estimates (*n* = 4). Twenty-four studies met the inclusion criteria and were retained for qualitative synthesis. Five studies were excluded from quantitative synthesis due to incompatible outcome metrics or incomplete data. Nineteen studies were included in the meta-analysis. Fig. 1 illustrates the PRISMA flow diagram of the study selection and literature screening process.

### Study characteristics

The final sample included twenty case-control studies and four cohort studies. All included studies investigated the association between exposure to diagnostic dental radiography and the development of various cancers. The imaging modalities assessed included full-mouth periapical radiographs, bitewings, panoramic, and cephalometric X-rays [14–17, 19–23, 28]. Three studies involved head CT scans [24–26]. However, some studies did not specify the exact type of dental radiograph performed [27-37]. Evaluated outcomes comprised CNS tumors, thyroid cancer, lymphoid cancer, oral cancer, salivary gland cancer, and breast cancer.

The bibliometric characteristics of the included studies are presented in Table 1.

**Table 1:** Summary of study characteristics

Author/country/ year	Study design	Sample size	Mean follow up	Exclusion period before a cancer diagnosis	Dental x-ray types	Cancer type	Confounding Adjustment	Effect Estimate and Interpretation
Burch et al., (Canada) 1987 [27]	Case-control	*N* = 430. G1 (cases) *n* = 215. G2 (controls) *n* = 215.	/	Exposure assessed before diagnosis	Any dental X-ray (unspecified)	Brain tumor	Age, gender, marital status	No association. (RR = 1.25; *p* = 1.00)
Nishi and Miyake, (Japan) 1989 [28]	Case-control	*N* = 189. G1 (cases) *n* = 63. G2 (controls) *n* = 126.	/	None (young children)	Any dental X-ray (unspecified)	Non-T Cell Acute Lymphoblastic leukaemia	BCG vaccination, measles infection or vaccination, asthma, atopic dermatitis, hip radiography, maternal milk consumption during pregnancy, and animal contact.	Positive association. (RR = 1.4, 95% CI = 1.0–2.0 ; *p* < 0.05)
Preston-Martin et al., (USA) 1989 [14]	Case-control	*N* = 544. G1 (cases with glioma) *n* = 202. G2 (cases with meningioma) *n* = 70. G3 (controls) *n* = 272.	/	2 years	Full-mouth dental X-rays	Glioma and meningioma	Occupational factors, tobacco and alcohol	No association. Glioma (OR = 3; 95% CI = 0.6–14.9) Meningioma OR = 2.5; CI = 0.2–29.5) No statistical significance
Ryan et al., (Australia) 1992 [15]	Case-control	*N* = 587. G1 (cases) G1 (cases with glioma) *n* = 110. G2 (cases with meningioma) *n* = 60. G3 (controls) *n* = 417.	/	2 years	Panoramic and Intraoral (Full-mouth)	Glioma and meningioma	Age, gender	No association. Glioma (RR = 0.42; 95% CI = 0.24–0.76) Meningioma (RR = 1.37; CI = 0.68–2.73)
Wingren et al., (Swede) 1993 [29]	Case-control	*N* = 491. G1 (cases) *n* = 104. G2 (controls) *n* = 387.	/	5 years	Any dental X-ray (unspecified)	Papillary thyroid cancer	Age, geographic area, and other covariates	Positive association. Multiple exposures (OR = 2.8 ;95%CI = 1.1–7.5)
Hallquist et al., (Sweden) 1994 [30]	Case-control	*N* = 540. G1 (cases) *n* = 180. G2 (controls) *n* = 360.	/	5 years	Any dental X-ray (unspecified)	Thyroid cancer	Age, gender	Positive association for multiple exposures OR = 2.4; 95%CI = 1.0–8.0) Increase in risk for women (OR = 4.8; 95% CI = 1.6–19)
Inskip et al., (Sweden) 1995 [16]	Case-control	*N* = 968. G1 (cases) *n* = 484. G2 (controls) *n* = 484.	/	5 years	Panoramic and Intraoral (Full-mouth)	Thyroid cancer	Age, gender, and geographic area.	No association. (RR = 2.3; 95% CI = 1.1–4.8)
Horn-Ross et al., (USA) 1997 [17]	Case-control	*N* = 332. G1 (cases) *n* = 141. G2 (controls) *n* = 191.	/	Exposure assessed before diagnosis	Intraoral (Full-mouth)	Salivary gland cancer	Age, gender, and other potential confounders	Positive association. (OR = 1.6; 95% CI = 1.0–2.7)
Schildt et al., (Sweden) 1998 [31]	Case-control	*N* = 708. G1 (cases) *n* = 354. G2 (controls) *n* = 354.	/	Exposure assessed before diagnosis	Any dental X-ray (unspecified)	Oral cancer	Age, gender, and other potential confounders	No association. (OR = 1.4; 95% CI = 0.8–2.6)
Rodvall et al., (Sweden) 1998 [32]	Case-control	*N* = 617. G1 (cases) *n* = 274. G2 (controls) *n* = 343.	/	Exposure assessed before diagnosis	Any dental X-ray (unspecified)	CNS tumors	Age, gender	No association (RR = 1.1 ; 95% CI = 0.7–1.9) Meningioma RR = 2.1; 95% CI = 1.0-4.3
Longstreth et al., (USA) 2004 [18]	Case-control	*N* = 600. G1 (cases) *n* = 200. G2 (controls) *n* = 400.	/	10 years	Panoramic, cephalometric and intraoral (bitewing/periapical)	Meningioma	Gender and education.	Positive association for ≥ 6 full-mouth series (OR = 2.06; 95% CI = 1.03–4.17) No association for other dental X-rays.
Ruder et al., (USA) 2006 [19]	Case-control	*N* = 1,673. G1 (cases) *n* = 498. G2 (controls) *n* = 1,175.	/	Exposure assessed before diagnosis	Panoramic	Glioma	Age, gender, education.	No association. (OR = 0.67; 95% CI = 0.52–0.86)
Ma et al., (USA) 2008 [33]	Case-control	*N* = 2,183. G1 (cases) *n* = 1,742. G2 (controls) *n* = 441.	/	5 years	Any dental X-ray (unspecified)	Breast cancer	Age, race, social economic status and residence.	Positive association for exposure before age of 20 and no lead apron (OR = 1.8; 95% CI = 1.13–2.90)
Memon A et al., (Kuwait) 2010 [34]	Case-control	*N* = 626. G1 (cases) *n* = 313. G2 (controls) *n* = 313.	/	Exposure assessed before diagnosis	Any dental X-ray (unspecified)	Thyroid cancer	Age, nationality, residence, upper-body X-rays	Positive association (OR = 2.1, 95% CI = 1.4–3.1) OR for multiple exposures: (10+) = 5.4 (95% CI = 1.1–26.7)
Davis F et al., (US) 2011 [35]	Case-control	*N* = 538. G1 (cases) *n* = 205. G2 (controls) *n* = 333.	/	2 years	Panoramic and unspecified intraoral dental X-ray	Glioma	Age, gender, family history of cancer, Socioeconomic status	No association for dental x-ray (OR = 0.6, 95% CI = 0.21–1.73)
Han Y-Y et al., (US) 2012 [20]	Case-control	*N* = 686. G1 (cases) *n* = 343. G2 (controls) *n* = 343.	/	Exposure assessed before diagnosis	Panoramic and Intraoral (periapical)	Vestibular schwannoma	Race, education, smoking, alcohol, noise exposure, cell phone use, diabetes, family history of cancer	Positive association (OR = 4.26, 95% CI = 1.49–12.18)
Claus EB et al., (US) 2012 [21]	Case-control	*N* = 2,783. G1 (cases) *n* = 1,433. G2 (controls) *n* = 1,350.	/	Exposure assessed before diagnosis	Panoramic and intraoral (bitewing/periapical)	Meningioma	Age, gender, education, race, head CT history	Positive association for bitewing: (OR = 2.0, 95% CI = 1.4–2.9)
Lin MC et al., (Taiwan) 2013 [22]	Case-control	*N* = 21,600. G1 (cases with BBT) *n* = 4,123. G2 (controls) *n* = 16,492. G3 (cases with MBT) *n* = 197. G4 (controls) *n* = 788.	/	Exposure assessed before diagnosis	Panoramic, cephalometric and intraoral (bitewing/periapical)	Benign and malignant brain tumor	Age, gender, dementia, epilepsy, Socioeconomic status	No significant association for malignant brain tumor (OR = 0.99, 95% CI = 0.69–1.44) Positive association for benign brain tumor (OR = 1.38, 95% CI = 1.28–1.48)
Neta et al., (US) 2013 [23]	Prospective cohort	*N* = 75,494. G1(cases) *n* = 251. G2 (non cases) *n* = 75,243.	17 years	5 years	Panoramic and Intraoral (bitewing/periapical)	Thyroid cancer	Gender, date of birth, smoking, BMI, thyroid history, occupational radiation	Positive association HR = 1.13, 95% CI = 1.01–1.26 Multiple exposures (30+): HR = 1.46, 95% CI = 0.93–2.30
Matthews J et al., (Australia) 2013 [24]	Retrospective cohort	*N* = 10,939,634. G1(cases) *n* = 60,674. G2 (non cases) *n* = 10,878,960.	9.5 years	5 and 10 years	CT scans of the facial bones	Any cancer diagnosis	Age, gender, date of birth	Positive association IRRs for all cancers = 1.14, 95% CI = 1.01–1.28 IRRs for thyroid cancer:1.53, 95% CI = 1.05–2.22
Huang et al., (Taiwan) 2014 [25]	Retrospective cohort	*N* = 112,086. G1(exposed cohort) *n* = 24,418. G2 (unexposed cohort) *n* = 97,668.	Up to 8 years	2 years	Head CT scan	Benign and malignant brain tumor, Leukemia	Age, gender, Socioeconomic status, Comorbidities	No association HR = 1.84, 95% CI = 0.64–5.29 Positive association for multiple exposures (Ct ≥ 3) HR = 5.4, 95% CI = 1.25–20.4
Parodi et al., (Italy) 2015 [36]	Case-control	*N* = 407. G1(cases) *n* = 169. G2 (controls) *n* = 238.	/	Exposure assessed before diagnosis	Any dental X-ray (unspecified)	Lymphoid cancer	Age, gender, smoking, education, radiation, pesticides, hydrocarbons	No clear association (OR = 1.2, 95% CI = 0.7-2.0)
Zhang et al., (US) 2015 [37]	Case-control	*N* = 960. G1(cases) *n* = 462. G2 (controls) *n* = 498.	/	5 years	Any dental X-ray (unspecified)	Thyroid cancer	Age, gender, education, thyroid history, BMI, alcohol, radiation treatment, X-ray exposure	No association (OR = 0.89, 95% CI = 0.61–1.30) Positive association for multiple exposures (> once a year): (OR = 2.2, 95% CI = 1.03–4.72)
Nordenskjöld et al., (Sweden) 2017 [26]	Retrospective cohort	*N* = 82,052. G1(exposed cohort) *n* = 18,388. G2 (unexposed cohort) *n* = 63,664.	Up to 37 years	5 and 10 years	Head CT scan	Meningioma	Age, gender, referral notes, radiology reports, exclusion of prevalent tumors, family history of cancer, Socioeconomic status	No clear association (HR = 1.49, 95% CI = 0.97–2.30)

*CI* Confidence Interval, *OR* Odds Ratio, *RR* Relative Risk, *HR* Hazard Ratio, *IRR* Incidence Rate Ratio, *CT* Computed Tomography, *BMI* Body Mass Index

### Methodological quality assessment

All studies showed adequate comparability between cases and controls, with appropriate matching, except for two which showed selection bias [19, 20]. In the study by Han Y-Y et al [20], controls were selected from patients attending an outpatient clinic for degenerative spinal disorders and had a higher frequency of CT scans, which may have introduced exposure misclassification and attenuated the observed association.

**Fig. 2:**
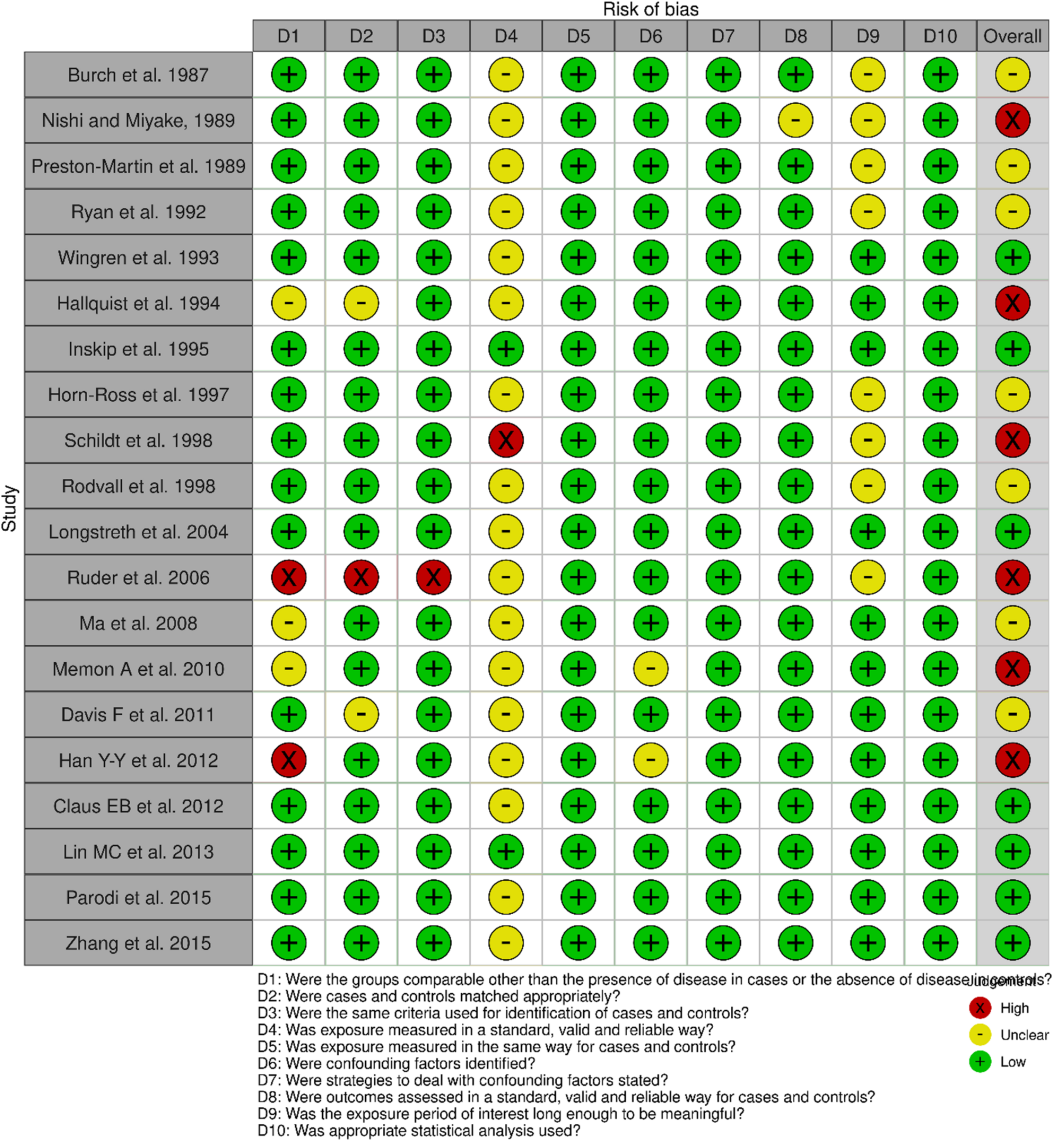
Critical appraisal results for included studies using the JBI-Critical Appraisal Checklist for case-control studies (traffic light plot)

**Fig. 3:**
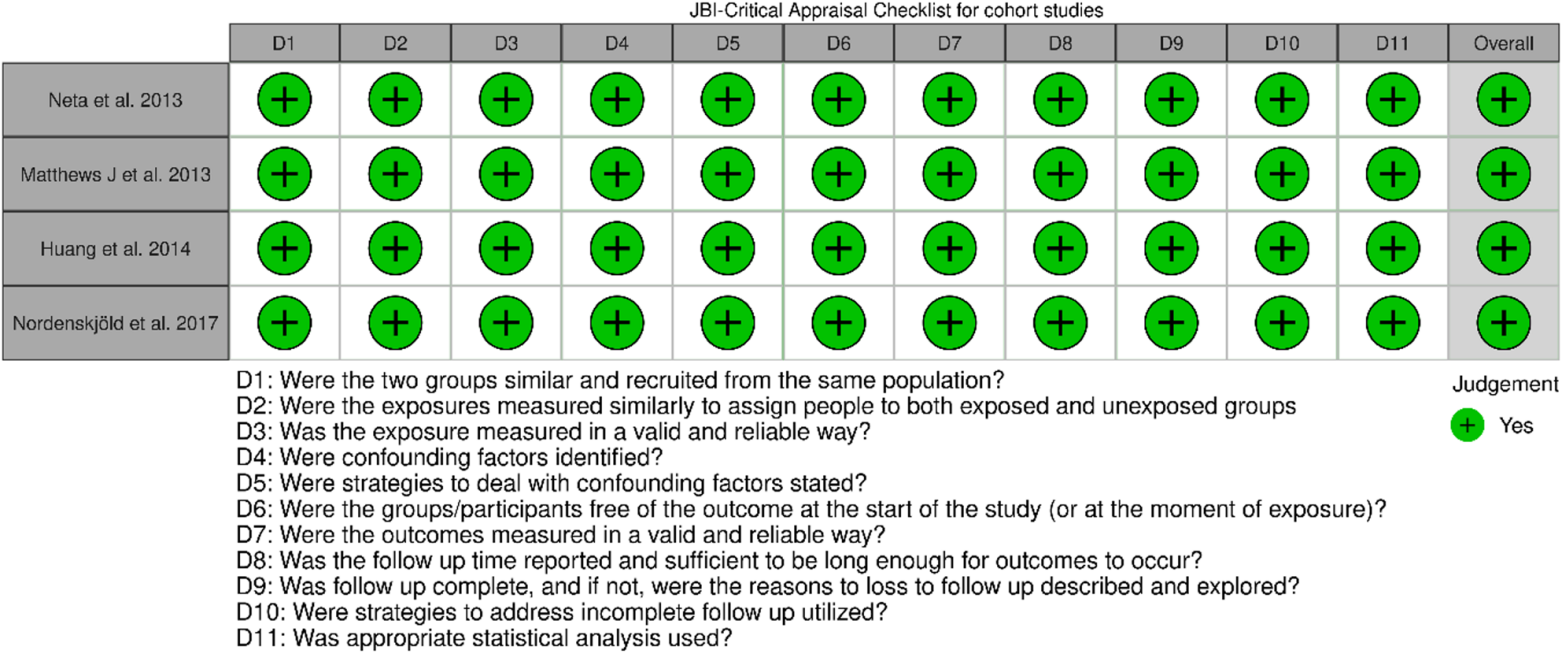
Critical appraisal results for included studies using the JBI-Critical Appraisal Checklist for cohort studies (traffic light plot)

Sixteen case-control studies [14–17, 19, 21, 22, 28–31, 33–37] and one cohort study [23] relied on self-reported dental X-ray exposure, introducing potential recall bias. Three cohort studies [24–26] and four case‒control study [18, 22, 27, 32] used registry-based data. Outcomes were confirmed through cancer registries or histopathological diagnosis. A summary of the findings is presented in Figs. 2 and 3.

Based on the COSMOS-E criteria, the strength of causal inference was graded as strong (*n* = 3), moderate (*n* = 14), or weak (*n* = 7), as presented in Table 2. Detailed grading for each study is presented in Table 2.

**Table 2:** Summary of causal inference grading according to COSMOS-E guidelines

Author	Confounding Control	Selection Bias	Information Bias	Study Sensitivity	Overall Grade
Burch et al. [27]	Low risk	Moderate risk (hospital-based)	Low risk (self-report and dental records)	Moderate risk	Moderate
Nishi and Miyake [28]	High risk	Moderate risk (hospital-based)	Moderate risk (self-report)	High risk	Weak
Preston-Martin et al. [14]	Low risk	Low (population-based cancer registry)	Moderate risk (self-report)	Moderate risk	Moderate
Ryan et al. [15]	Moderate risk	Low (Hospital-based and population-based cancer registry)	Moderate risk (self-report)	Moderate risk	Weak
Wingren et al. [29]	Low risk	Low (population-based cancer registry)	Moderate risk (self-report)	Low risk	Moderate
Hallquist et al. [30]	Moderate risk	Low (population-based cancer registry)	Moderate risk (self-report)	Low risk	Moderate
Inskip et al. [16]	Low risk	Low (population-based cancer registry)	Moderate risk (self-report)	Low risk	Moderate
Horn-Ross et al. [17]	Low risk	Low (population-based cancer registry)	Moderate risk (self-report)	Moderate risk	Moderate
Schildt et al. [31]	Low risk	Low (population-based cancer registry)	High risk (self-report with proxy interviews for deceased cases)	Moderate risk	Weak
Rodvall et al. [32]	Moderate risk	Low (Hospital-based and population-based cancer registry)	Low risk (self-report and dental records)	Moderate risk	Moderate
Longstreth et al. [18]	Moderate risk	Low (population-based cancer registry)	Low risk (self-report and dental records)	Low risk	Moderate
Ruder et al. [19]	Low risk	High risk (rapid case ascertainment through physicians and medical facilities)	Moderate risk (self-report)	High risk	Weak
Ma et al. [33]	Low risk	Low (population-based cancer registry)	Moderate risk (self-report)	Low risk	Moderate
Memon A et al. [34]	Moderate risk	Moderate risk (hospital-based)	Moderate risk (self-report)	High risk	Weak
Davis F et al. [35]	Low risk	Moderate risk (hospital-based and friend controls)	Moderate risk (self-report)	Moderate risk	Weak
Han Y-Y et al. [20]	High risk	High risk (hospital-based, spine disorders patient controls)	Moderate risk (self-report)	Low risk	Weak
Claus EB et al. [21]	Low risk	Low risk (population-based controls)	Moderate risk (self-report)	Moderate risk	Moderate
Lin MC et al. [22]	Low risk	Low risk (population-based)	Low risk (clinical databases and records)	Low risk	Strong
Neta et al. [23]	Low risk	Moderate risk (nationwide recruitment of certified radiologic technologists)	Moderate risk (self-report)	Low risk	Moderate
Matthews J et al. [24]	Moderate risk	Low risk (population-based)	Low risk (clinical databases and records)	Moderate risk	Moderate
Huang et al. [25]	Low risk	Low risk (population-based)	Low risk (clinical databases and records)	Low risk	Strong
Parodi et al. [36]	Low risk	Low risk (population-based)	Moderate risk (self-report)	Low risk	Moderate
Zhang et al. [37]	Low risk	Low risk (population-based)	Moderate risk (self-report)	Low risk	Moderate
Nordenskjöld et al. [26]	Low risk	Low risk (population-based)	Low risk (clinical databases and records)	Low risk	Strong

### Results of syntheses

Given the differences in effect measures across studies, meta-analyses were conducted separately for studies reporting hazard ratios (HRs) and those reporting odds ratios (ORs).

#### Meta-analysis of case-control studies

**Fig. 4:**
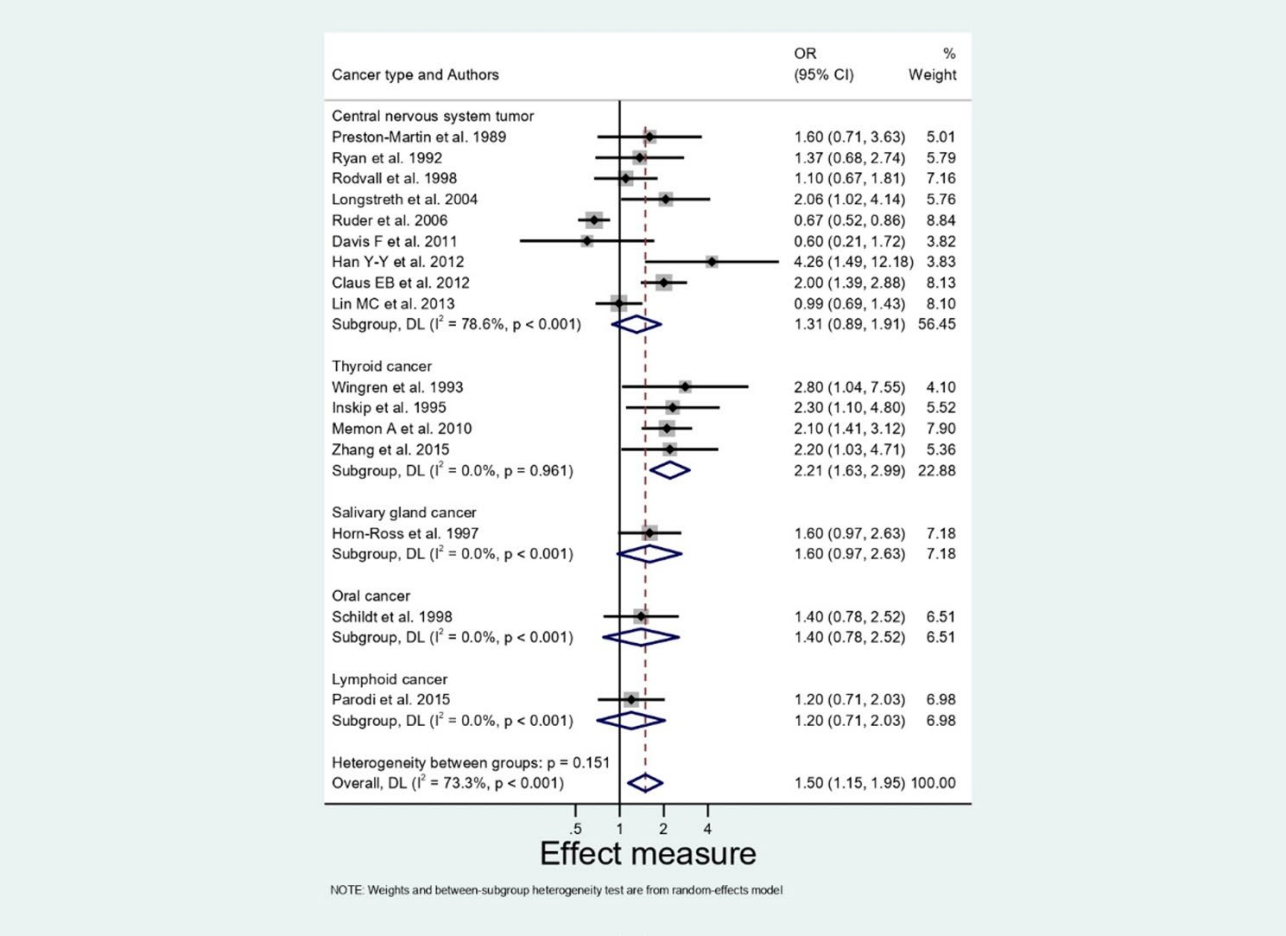
Forest plot of Odds ratios (ORs) by cancer type in case-control studies

Sixteen case-control studies were included in the quantitative synthesis. Substantial heterogeneity was observed between groups (*I*^*2*^ = 73.3%, *p* < 0.001). To account for the differing sensitivities and carcinogenic mechanisms among cancers, the pooled odds ratio was stratified by cancer type (Fig. 4):

- Central nervous system (CNS) tumors: Nine studies (*n* = 28,343; 3,338 incident cases) reported a pooled OR of 1.31 (95% CI: 0.89–1.91). The association was moderate, corresponding to a 31% increase in risk, but imprecise as the confidence interval included the null value. Substantial heterogeneity was present across studies (*I*² = 78.6%, *p* < 0.001).
- Thyroid cancer: Four studies (*n* = 3,092; 1,269 incident cases) yielded a pooled OR of 2.21 (95% CI: 1.63–2.99), indicating a significant association. No heterogeneity was observed across studies (*I²* = 0.0%, *p* = 0.961), suggesting consistency in the effect estimates.
- Lymphoid cancer: One study (*n* = 573; 169 incident cases) reported an OR of 1.20 (95% CI: 0.71–2.03).
- Salivary gland cancer: One study (*n* = 332; 139 incident cases) reported an OR of 1.60 (95% CI: 0.97–2.63).
- Oral cancer: One study (*n* = 708; 354 incident cases) reported an OR of 1.40 (95% CI: 0.78–2.52).

Although the estimate for lymphoid, oral and salivary gland cancer suggested increased risk, the limited number of contributing studies constrained certainty. Therefore, no conclusions can be drawn.

To explore sources of heterogeneity, studies were stratified by risk of bias using the JBI checklist and by recall bias. When stratified by risk of bias (Supplementary Fig. 1), the highest pooled estimate was observed among low-risk studies (*n* = 7) with a pooled OR of 1.68 (95% CI: 1.23–2.29), and moderate heterogeneity (*I*^*2*^ = 51.7%, *p* = 0.053).

When stratified by exposure ascertainment method (Supplementary Fig. 2), studies relying on self-reported dental imaging exposure (*n* = 13) yielded a pooled OR of 1.58 (95% CI: 1.14–2.19), while studies using medical or dental records for exposure ascertainment (*n* = 3) yielded a pooled OR of 1.20 (95% CI: 0.82–1.74).

Sensitivity analyses supported the robustness of the findings. A leave-one-out sensitivity analysis was conducted to assess the influence of individual studies on the pooled estimate (Supplementary Fig. 3). The Odds ratio remained statistically significant, ranging from 1.43 (95% CI: 1.10–1.86) after omitting Han et al. study [22], to 1.59 (95% CI: 1.30–1.94) after omitting Ruder et al. study [21].

All the case-control studies involved conventional dental radiography. However, stratification by radiographic modality (bitewing, periapical, panoramic, lateral cephalometric) was not conducted because of inconsistent exposure reporting. Several studies combined multiple imaging types or used nonspecific categories such as “any dental X-ray”, which could lead to exposure misclassification and reduce interpretability.

#### Meta-analysis of cohort studies

**Fig. 5:**
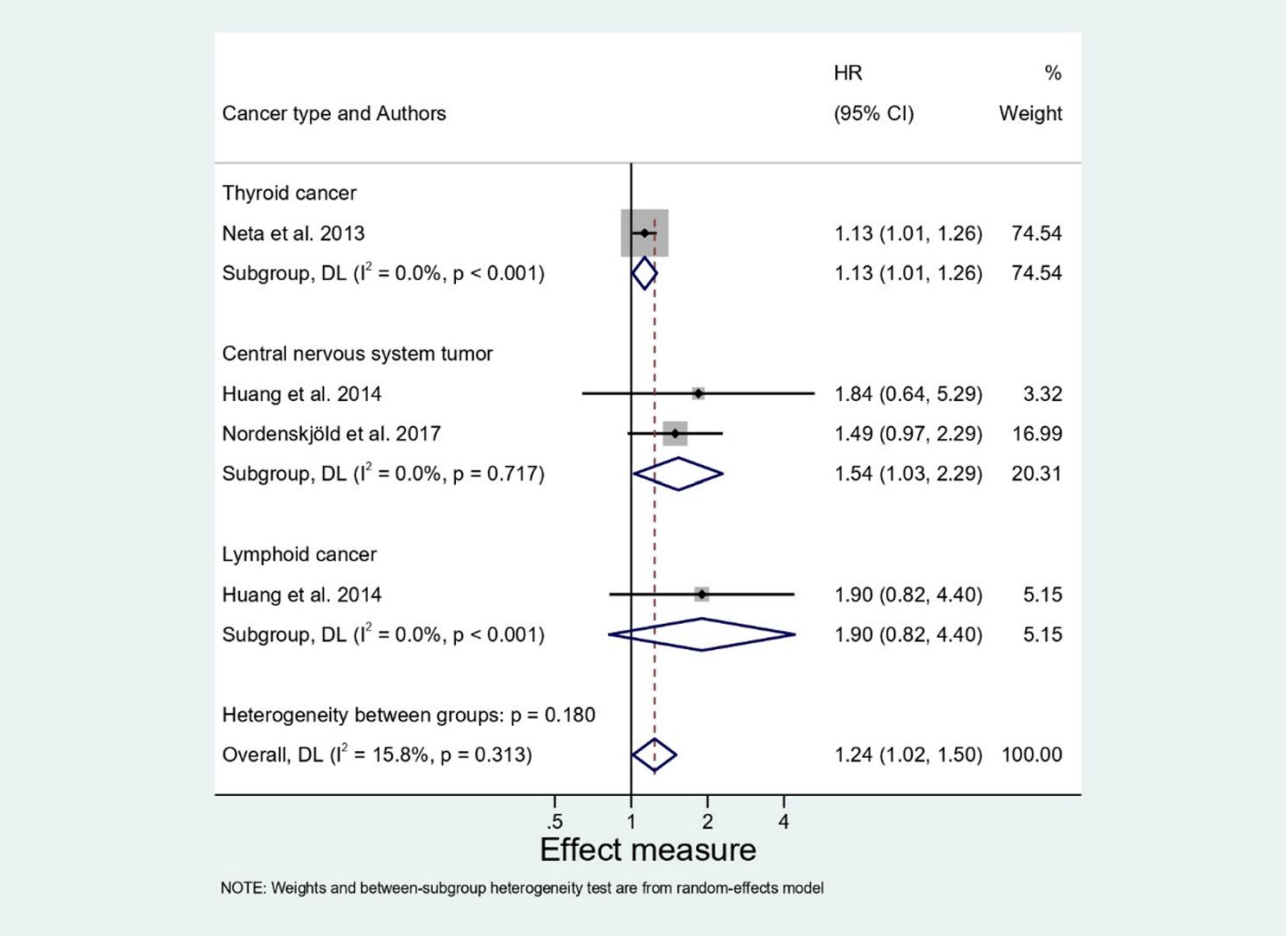
Forest plot of cancer risk by cancer type in cohort studies

Three cohort studies were included, with low heterogeneity overall (*I*^*2*^ = 15.8%, *p* = 0.313). Hazard Ratios were pooled and stratified by cancer type (Fig. 5):

- Thyroid cancer: One study based on conventional radiography (*n* = 75,494; 72,606 incident cases) yielded a HR of 1.13 (95% CI: 1.01–1.26), indicating a small but statistically significant association between conventional dental radiography and thyroid cancer risk.
- Central nervous system (CNS) tumors: Two studies (*n* = 194,138; 42,806 incident cases) reported a pooled HR of 1.54 (95% CI: 1.03–2.29). The association was statistically significant, and heterogeneity was negligible.
- Lymphoid cancer: One study (*n* = 112,086; 24,418 incident cases) reported an HR of 1.90 (95% CI: 0.82–4.40). The wide confidence interval, which includes the null value, indicates substantial imprecision, and the association was not statistically significant.

**Fig. 6:**
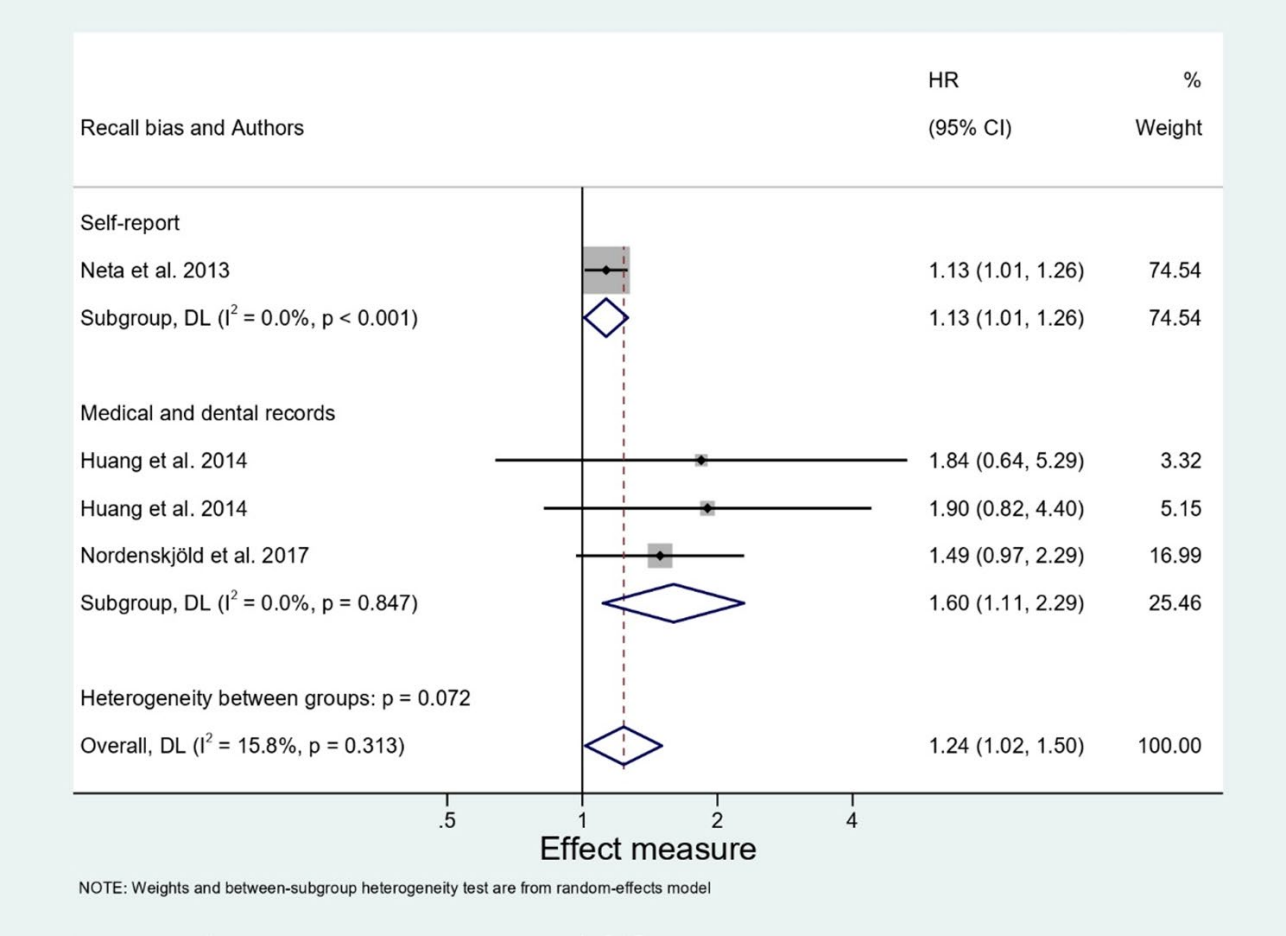
Forest plot of cancer risk stratified by radiographic modality in cohort studies

Both CNS and lymphoid cancer estimates were derived from CT scan exposures. When stratified by radiographic modality, conventional dental X-ray showed an HR of 1.13 (95% CI: 1.01–1.26), while CT scan exposure yielded a pooled HR of 1.60 (95% CI: 1.11–2.29; *I*^*2*^ = 0.0%) Subgroup differences by exposure modality and by exposure ascertainment method were not statistically significant (*p* = 0.072) (Fig. 6 and Supplementary Fig. 4).

A leave-one-out sensitivity analysis was conducted to assess the influence of individual studies (Supplementary Fig. 5). The pooled HR remained relatively stable, but the small number of cohort studies (*n* = 3), the results of the should be interpreted as exploratory .

Publication bias was present for case-control studies. The funnel plot appeared visually asymmetrical (Supplementary Fig. 6), and Egger’s test was significant (*p* = 0.023), indicating a small-study effect. Publication bias was not assessed for cohort studies due to the insufficient number of included studies (*n* = 3).

### Certainty of evidence

The certainty of evidence was rated low for thyroid cancer, and very low for all other outcomes. These findings suggest that the true effects may differ substantially from the observed estimates. For thyroid cancer and central nervous system cancers, the evidence points to a small positive association, but it is imprecise and inconsistent across studies. A summary of certainty ratings is presented in Table 3.

**Table 3:** Certainty of evidence (GRADE analysis)

Outcome	Number of studies	Study design	Risk of Bias	Inconsistency (I²)	Indirectness	Imprecision (95% CI width)	Other Considerations	Certainty	Importance
Thyroid cancer risk	5	Case-control/cohort	Serious *	Not serious	Not serious	Not Serious	Dose-response present (5 studies)	Low ⊕⊕⊖⊖	Critical
CNS tumors	11	Case-control/cohort	Serious *	Very Serious **	Not serious	Serious §	Dose-response present (11 studies)	Very low ⊕⊖⊖⊖	Critical
Lymphoid cancer	2	Case-control/cohort	Not serious *	Not serious	Not serious	Serious §	Limited number of studies	Very low ⊕⊖⊖⊖	Important
Oral cancer	1	Case-control	Very serious *	Not serious	Not serious	Serious §	Limited number of studies	Very low ⊕⊖⊖⊖	Important
Salivary gland cancer	1	Case-control	Serious *	Not serious	Not serious	Serious §	Limited number of studies	Very low ⊕⊖⊖⊖	Important

Other Considerations: Dose-response trends, large effect sizes, or plausible confounding that would reduce observed associations were considered for upgrading

Critical outcomes were considered essential for clinical decision-making; Important outcomes informed secondary interpretations. Certainty of evidence levels reflect the likelihood that the true effect differs substantially from the estimate

* Risk of Bias assessed using JBI checklists for observational studies and COSMOS-E framework for causal inference. Downgrading was applied if one or more domains demonstrated serious limitations

** Downgraded based on the I² statistic. Thresholds applied were: 50–75% = serious inconsistency; >75% = very serious inconsistency

‡ Downgraded for issues in population, exposure definition, or cancer outcome specificity

§ Imprecision was rated serious if: (a) 95% confidence interval includes the null value (OR = 1), or (b) total number of events < 300

According to the World Cancer Research Fund (WCRF) Global Cancer Update Program grading criteria [38], this evidence is best classified as limited–no conclusion.

## Discussion

### Summary of key findings

Across study designs, thyroid cancer showed a consistent association with conventional dental imaging, with a significant association in both cohort and case-control studies. Central nervous system tumors were associated with CT scan exposure in cohort studies, while case-control studies showed an elevated but non-significant association. These findings are biologically plausible, given the proximity of the thyroid gland to the primary X-ray scatter field and the known radiosensitivity of neural tissues. However, the certainty of evidence was rated low for thyroid cancer, and very low for central nervous system cancers.

### Thyroid cancer

Thyroid cancer is among the most prevalent cancers globally. This systematic review included seven studies published between 1993 and 2015 examining thyroid cancer risk associated with low dose ionizing radiation from dental practice. The meta-analysis demonstrated an elevated thyroid cancer risk in both case-control studies (OR = 2.21; 95% CI: 1.63–2.99) and one cohort study (HR = 1.13; 95% CI: 1.01–1.26) [16, 23, 34, 37, 39]. Both study designs relied on self-reported exposure, but the direction of bias differs: the stronger association in case-control studies is likely due to recall bias, as exposure was ascertained after diagnosis, possibly resulting in cases reporting dental X-rays more frequently than controls. The prospective cohort study’s smaller effect estimate may be explained by non-differential misclassification, which attenuates effect estimates toward the null. Additionally, the cohort’s larger sample size provides greater statistical precision. Overall, these findings are consistent with a previous meta-analysis that reported elevated risks for thyroid cancer (pooled RR = 1.87, 95% CI: 1.11–3.15) [39].

When interpreting the findings, it’s important to consider variations in radiation doses across imaging modalities and time periods. A study conducted during periods of historically higher radiation found no association with thyroid cancer following a 5-year exclusion period [16].

Five studies reported a positive association with thyroid cancer [23, 30, 31, 34, 37], including two conducted during the same historical period [29, 30]. Furthermore, the study by Zhang and al found a positive association only with multiple exposure [37]. This suggests that the cumulative effect from repeated exposures could explain the difference in outcomes, as early studies did not account for this factor. Although a dose response relationship is observed [34, 37], it cannot be confirmed as the studies did not consistently provide data on the nature of the dental radiographs performed and the precise definition of multiple exposure.

Thyroid doses from dental radiography have decreased markedly over the decades following advances in imaging radiology. However, the risk association remains. The carcinogenic effect of radiation is complex and interplays with factors such as age at exposure, frequency of exposure, lifetime prevalence, and the use of thyroid shielding [40].

Computed tomography provides additional context for assessing risk related to low-dose ionizing radiation. As defined by the ICRP, LDIR encompasses diagnostic procedures with an effective dose ≤ 100 mSv [41]. For context, the average effective dose for a single periapical radiograph ranges from 0.001 to 0.008 mSv, effective dose for panoramic radiograph ranges from 0.004 to 0.030 mSv, while CBCT effective dose ranges from 0.050 to 0.550 mSv [42]. These dental exposures fall within the very low to minimal dose range following ICRP (2021) terminology [41]. Radiation from CT scans of the skull or facial bones can range from 0.5 to 1 mSv [42]. Although relatively higher, it remains within the very low category. A cohort study reported a partial association between CT scan exposure of the facial bones and increased thyroid cancer risk. However, this analysis did not adjust for concurrent dental radiographs [24].

### Central nervous system tumors

Ionizing radiation is an established environmental risk factor for CNS tumor. Nonetheless, the effect of low-dose ionizing radiation from dental imaging remains unclear [2]. Other risk factors include a history of brain injury, smoking, chronic virus infection, and occupational exposure.

Regarding meningioma, an increased risk was reported for bitewing and panoramic radiographs, with up to fivefold increase for individuals exposed before age 10 [21]. Another study found an association limited to repeated full-mouth series (6 over a lifetime), without evidence for a dose-response relation [18]. However, substantial heterogeneity was found among studies, as several showed no association [14, 15, 32]. This heterogeneity may partly reflect variations in radiation technology and dosimetry across decades. Older studies often involved higher-dose D-speed film and full-mouth series, whereas more recent practice uses lower-dose F-speed film and digital sensors. Additionally, differences in exposure parameters likely contribute to the observed inconsistency.

A previous meta-analysis review concluded that there is no association between exposure to dental X-rays and the risk of development of meningioma (OR = 0.97; 95% CI, 0.70–1.32). The same analysis found that exposure to bitewing X-rays was associated with a small increase in risk (OR = 1.73; 95% CI, 1.28–2.34) [43].

The evidence for gliomas is more consistent. Four studies on panoramic and full-mouth radiograph showed no association for glioma [14, 15, 19–35]. Notably, these studies did not assess childhood exposure, a critical period of vulnerability. In contrast, an increased risk of benign brain tumors was observed, particularly in children aged 0–6 years, but no association was found for malignant tumors [22].

A study linking childhood CT exposure to brain tumors reported a 2.6-fold increased risk in children and adolescents undergoing head CT scans, with incidence increasing with repeated scans, particularly in genetically predisposed individuals [25]. Importantly, these risk estimates are based on higher radiation doses from CT scans performed between 1985 and 2005. In contrast, a cohort of over 15,000 individuals exposed to head CT scans with a follow-up of 37 years reported no significant increase in meningioma risk, although it did not specifically examine childhood exposure [26].

A French cohort study further questioned the link between CT scan and childhood cancer risk. they reported no significant associations after adjusting for predisposing factors, emphasizing the need to consider clinical indications for imaging, as reverse causality or confounding bias may lead to overestimated risk estimates [44].

While only one study associated historical dental X-ray exposure with vestibular schwannoma [20], it’s finding are limited by critical methodological flaws. An observed inverse association with head CT scans likely reflects selection bias, as controls may have received scans for underlying neurological conditions.

### Lymphoid cancer

A limitation of this review is the inability to differentiate risks between lymphoid and myeloid leukemia types. One study did not find an association with dental X-ray exposure and lymphoid cancer after adjusting for confounders [36]. In contrast, an older study that found a positive association for non t-cell acute lymphoblastic leukaemia [28]. Despite an elevated odds ratio for children aged 3–6 years, an association with dental X-rays alone could not be established, as no association was found for the 7–14 year age group where dental X-rays are frequently performed [28].

In a pooled analysis of CT scans and childhood risk of cancer, increased risks of both acute lymphoid leukaemia and acute myeloid leukaemia were reported at cumulative doses less than 100 mSv during childhood or adolescence, with dose-response relationships remaining significant at doses less than 50 mSv [45].

### Other cancers

This systematic review identified several studies on diverse cancer outcomes. A positive association was found for salivary gland cancer, while another study reported no association for oral cancer [17–31]. This discrepancy may reflect differences in organs susceptibility and methodological limitations. A positive association was observed for breast cancer in women exposed before age 20 without lead apron use [33]. This finding suggests a potential role for age at exposure and shielding practices, but cannot establish a direct effect of dental radiation. Overall, the high heterogeneity across these studies overrides definitive conclusions.

Two studies were excluded from the systematic review due to serious methodological limitations, particularly the reporting of unadjusted risk estimates. The first linked historical dental X-ray exposure and heavy smoking to laryngeal cancer, but found no association for light smokers [46]. The second found a positive risk association with parotid gland tumors, both benign and malignant [47]. In both cases, the observed effect could not be attributed to dental X-ray exposure alone. Without adjustment for potential confounders, such findings represent an oversimplification of the causal relationship and may not reflect the true association.

A systematic review [48], a comprehensive review [49], and two meta-analyses [39–43] examined the biological and health effects of dental radiography. The first systematic review [48] reported potential associations between meningioma and thyroid cancer, although the conclusions were limited by sparse data. Similarly, the comprehensive review [49] reported that while epidemiological studies suggest a link between dental X-rays and head/neck tumors, causal evidence remains insufficient. The results of their biomonitoring analyses revealed localized cytotoxic effects from low-dose exposure but no persistent chromosomal damage [49]. The lack of dose-stratified data and inability to establish causation prevents definitive conclusions, mirroring the limitations identified in our meta-analysis.

Detecting radiation-associated cancer at low doses remains inherently challenging, even in large-scale epidemiological studies. While radiation dose serves as a surrogate for risk estimation in the LNT model, there is no direct evidence of effects at doses of the order of 10 mSv and below because of the limitations imposed by statistical uncertainties in identifying possible small excesses of disease above background levels [50]. Additionally, the prolonged latency period between exposure and cancer development, combined with the complex interplay of genetic and environmental factors in carcinogenesis, makes distinguishing radiation-induced malignancies from spontaneously occurring cases difficult [49].

Our findings are consistent with the principle that epidemiologic associations alone are insufficient for establishing causality in cancer research. Incorporation of mechanistic evidence, such as biomarker and cellular studies, is crucial when interpreting exposure-cancer relationships [51].

### Strengths and limitations

The strengths of this review lie in its comprehensive analysis of epidemiological evidence linking LDIR from dental imaging to cancer risk, with all pooled estimates derived from multivariate-adjusted Hazard Ratios and Odds Ratios. This approach addresses a limitation of prior pooled analyses, which included studies with unadjusted risk estimates, thereby supporting more reliable causal inference. The review spans evidence from 1987 to the present, capturing both historical and contemporary imaging practices.

Several limitations should be considered. Methodological heterogeneity existed across studies due to varying data collection methods and inconsistent exposure reporting, introducing recall bias. Confounding by indication in the CT studies limited interpretation. Differences in follow-up durations and control for confounding factor further contributed to the observed heterogeneity. Additionally, defining radiation exposure was challenging owing to differences in dental imaging equipment, exposure parameters, shielding practices, and technological improvements over time. Consequently, no dose-response relationship can be established. Furthermore, publication bias assessment was limited by the small number of studies included, rendering statistical tests underpowered and requiring cautious interpretation.

Another limitation is the lack of cone-beam computed tomography (CBCT) specific epidemiological data. Current evidence relies on dosimetry modeling, suggesting increased radiosensitivity in pediatric and female populations (particularly for thyroid and oral cancers) [52]. Although individual cancer risk estimates are small, the concern about risks from CBCT relates to the rapid increase in its use for orthodontic practice, especially in children [53].

### Clinical and research implications

Given the persistent uncertainties in low-dose radiation risk assessment, rigorous application of the ALADAIP principle (as low as diagnostically acceptable, indication-oriented and patient-specific) [54] is essential. Advances in imaging technology, such as rectangular collimation and digital sensors, have led to significant reductions in patient exposure without compromising diagnostic quality [55]. To strengthen the evidence base, future studies should (1) provide large-scale prospective cohorts with precise dosimetry and adequate follow-up; (2) systematically adjust for relevant confounding factors; and (3) include all dental imaging modalities with particular emphasis on CBCT given its growing clinical use to allow accurate dose-response characterization across imaging techniques.

## Conclusion

Evidence on cancer risk from low-dose dental radiographic exposure remains inconclusive. The certainty of the evidence is very low, and a causal relationship cannot be confirmed. The biological plausibility highlights the importance of radiation protection. Thus, given the increasing frequency of radiographs and the growing shift toward 3D imaging, adherence to the ALADAIP principle (as low as diagnostically acceptable, indication-oriented and patient-specific) is critical and would support future research in refining radiation risk assessment in current clinical practice.

## Supplementary Information

Supplementary Material 1: Figure 1: Forest plot of cancer risk stratified by risk of bias in case-control studies.

Supplementary Material 2: Figure 2: Forest plot of cancer risk stratified by exposure ascertainment method.

Supplementary Material 3: Figure 3: Leave-one-out sensitivity analysis for case-control studies.

Supplementary Material 4: Figure 4: Forest plot of cancer risk stratified by exposure ascertainment method cohort studies.

Supplementary Material 5: Figure 5: Leave-one-out sensitivity analysis for cohort studies.

Supplementary Material 6: Figure 6: Funnel plot for publication bias assessment in case-control studies.

## Data Availability

All data generated or analyzed in this study are included in this published article and its supplementary files.

## Abbreviations

CT: Computed tomography
LNT: Linear no-threshold
IRSN: Institute for Radiation Protection and Nuclear Safety
LDIR: Low-dose ionizing radiation
CBCT: Cone-beam computed tomography
PRISMA: Preferred Reporting Items of Systematic Reviews and Meta-Analysis
PROSPERO: International prospective register of systematic reviews
PICO: Patient, Intervention, Comparator, Outcomes
OR: Odds Ratio
RR: Relative Risk
HR: Hazard Ratio
IRR: Incidence Rate Ratio
CIs: Confidence intervals
RoB: Risk of bias
JBI: Joanna Briggs Institute
COSMOS-E: Conducting Systematic reviews and Meta-analyses of Observational Studies of Etiology
CNS: Central nervous system
GRADE: Grading of Recommendations Assessment, Development and Evaluation
BMI: Body Mass Index
WCRF: World Cancer Research Fund

## References

[1] Sources UNSCEAR, effects and risks of ionizing radiation. UNSCEAR 2020/2021 report to the general assembly, with scientific annexes. Volume I. Scientific annex A. Evaluation of medical exposure to ionizing radiation. New York: United Nations Scientific Committee on the Effects of Atomic Radiation. 2022. Available from: https://www.unscear.org/unscear/uploads/documents/publications/UNSCEAR_2020_21_Annex-A.pdf.

[2] UNSCEAR 2021 sources, effects and risks of ionizing radiation. UNSCEAR 2020/2021 report to the general assembly, with scientific annexes. Volume III. Scientific Annex C. Biological mechanisms relevant for the inference of cancer risks from low-dose and low-dose-rate radiation (New York: United Nations Scientific Committee on the Effects of Atomic Radiation) Available from: https://www.unscear.org/unscear/uploads/documents/unscear-reports/UNSCEAR_2020_21_Report_Vol.III-CORR.pdf.

[3] UNSCEAR. Report of the united nations scientific committee on the effects of atomic radiation. UNSCEAR 2013 Report. Volume II. Scientific annex B-effects of radiation exposure of children. United Nations. 2013. Available from: https://www.unscear.org/unscear/uploads/documents/publications/UNSCEAR_2013_Annex-B.pdf.

[4] Laurier D, Billarand Y, Klokov D, Leuraud K. The scientific basis for the use of the linear no-threshold (LNT) model at low doses and dose rates in radiological protection. J Radiol Prot. 2023;43(2). 10.1088/1361-6498/acdfd7. 10.1088/1361-6498/acdfd7 37339605

[5] Grant EJ, Brenner A, Sugiyama H, et al. Solid cancer incidence among the life span study of atomic bomb survivors: 1958–2009. Radiat Res. 2017;187:513–37. 10.1667/RR14492.1. 28319463 10.1667/RR14492.1 PMC10320812

[6] National Research Council. Health risks from exposure to low levels of ionizing radiation: BEIR VII Phase 2. Washington, DC: The National Academies Press. 2006. (Available from: 10.17226/11340 ).

[7] Page MJ, McKenzie JE, Bossuyt PM, et al. The PRISMA 2020 statement: an updated guideline for reporting systematic reviews. BMJ. 2021;372:n71. 10.1136/bmj.n71. 33782057 10.1136/bmj.n71 PMC8005924

[8] Munn Z, Stone JC, Aromataris E, et al. Assessing the risk of bias of quantitative analytical studies: introducing the vision for critical appraisal within JBI systematic reviews. JBI Evid Synth. 2023;21(3):467–71. 10.11124/JBIES-22-00224. 36476419 10.11124/JBIES-22-00224

[9] Barker TH, Hasanoff S, Aromataris E, et al. The revised JBI critical appraisal tool for the assessment of risk of bias for cohort studies. JBI Evid Synth. 2024. 10.11124/JBIES-24-00103. 39177422 10.11124/JBIES-24-00103

[10] McGuinness LA, Higgins JPT. Risk-of-bias VISualization (robvis): an R package and shiny web app for visualizing risk-of-bias assessments. Res Syn Meth. 2020;1–7. 10.1002/jrsm.1411. 10.1002/jrsm.1411 32336025

[11] Dekkers OM, Vandenbroucke JP, Cevallos M, et al. COSMOS-E: guidance on conducting systematic reviews and meta-analyses of observational studies of etiology. PLoS Med. 2019;16(2):e1002742. 10.1371/journal.pmed.1002742. 30789892 10.1371/journal.pmed.1002742 PMC6383865

[12] DerSimonian R, Laird N. Meta-analysis in clinical trials. Control Clin Trials. 1986;7(3):177–88. 10.1016/0197-2456(86)90046-2. 3802833 10.1016/0197-2456(86)90046-2

[13] Prasad M. Introduction to the GRADE tool for rating certainty in evidence and recommendations. Clin Epidemiol Glob Health. 2023;25:101484. 10.1016/j.cegh.2023.101484.

[14] Preston-Martin S, Mack W, Henderson BE. Risk factors for gliomas and meningiomas in males in Los Angeles County. Cancer Res. 1989;49(21):6137-43. PMID: 2790826. 2790826

[15] Ryan P, Lee MW, North B, McMichael AJ. Amalgam fillings, diagnostic dental x-rays and tumours of the brain and meninges. Eur J Cancer B Oral Oncol. 1992;28B(2):91 – 5. 10.1016/0964-1955(92)90034-x. PMID: 1306734. 10.1016/0964-1955(92)90034-x 1306734

[16] Inskip PD, Ekbom A, Galanti MR, Grimelius L, Boice JD Jr. Medical diagnostic x rays and thyroid cancer. J Natl Cancer Inst. 1995;87(21):1613-21. 10.1093/jnci/87.21.1613. PMID: 7563204. 10.1093/jnci/87.21.1613 7563204

[17] Horn-Ross PL, Ljung BM, Morrow M. Environmental factors and the risk of salivary gland cancer. Epidemiology. 1997;8(4):414-9. 10.1097/00001648-199707000-00011. PMID: 9209856. 10.1097/00001648-199707000-00011 9209856

[18] Longstreth WT Jr, Phillips LE, Drangsholt M, Koepsell TD, Custer BS, Gehrels JA, van Belle G. Dental X-rays and the risk of intracranial meningioma: a population-based case-control study. Cancer. 2004;100(5):1026-34. 10.1002/cncr.20036. PMID: 14983499. 10.1002/cncr.20036 14983499

[19] Ruder AM, Waters MA, Carreón T, Butler MA, Davis-King KE, Calvert GM, Schulte PA, Ward EM, Connally LB, Lu J, Wall D, Zivkovich Z, Heineman EF, Mandel JS, Morton RF, Reding DJ, Rosenman KD, Brain Cancer Collaborative Study Group. The upper midwest health study: a case-control study of primary intracranial gliomas in farm and rural residents. J Agric Saf Health. 2006;12(4):255 – 74. 10.13031/2013.22013. PMID: 17131948. 10.13031/2013.22013 17131948

[20] Han YY, Berkowitz O, Talbott E, Kondziolka D, Donovan M, Lunsford LD. Are frequent dental x-ray examinations associated with increased risk of vestibular schwannoma? J Neurosurg. 2012;117(Suppl):78–83. 10.3171/2012.5.GKS12615. 23211211 10.3171/2012.5.GKS12615

[21] Claus EB, Calvocoressi L, Bondy ML, Schildkraut JM, Wiemels JL, Wrensch M. Dental x-rays and risk of meningioma. Cancer. 2012;118(18):4530–7. 10.1002/cncr.26625. 22492363 10.1002/cncr.26625 PMC3396782

[22] Lin M-C, Lee C, Lin C, et al. Dental diagnostic X-ray exposure and risk of benign and malignant brain tumors. Ann Oncol. 2013;24(6):1675–9. 10.1093/annonc/mdt016. 23406732 10.1093/annonc/mdt016

[23] Neta G, Rajaraman P, Berrington de Gonzalez A, Doody MM, Alexander BH, Preston D, Simon SL, Melo D, Miller J, Freedman DM, Linet MS, Sigurdson AJ. A prospective study of medical diagnostic radiography and risk of thyroid cancer. Am J Epidemiol. 2013;177(8):800–9. 10.1093/aje/kws315. 23529772 10.1093/aje/kws315 PMC3668423

[24] Mathews JD, Forsythe AV, Brady Z, Butler MW, Goergen SK, Byrnes GB, Giles GG, Wallace AB, Anderson PR, Guiver TA, McGale P, Cain TM, Dowty JG, Bickerstaffe AC, Darby SC. Cancer risk in 680,000 people exposed to computed tomography scans in childhood or adolescence: data linkage study of 11 million Australians. BMJ. 2013;346:f2360. 10.1136/bmj.f2360. 23694687 10.1136/bmj.f2360 PMC3660619

[25] Huang WY, Muo CH, Lin CY, et al. Paediatric head CT scan and subsequent risk of malignancy and benign brain tumour: a nation-wide population-based cohort study. Br J Cancer. 2014;110:2354–60. 10.1038/bjc.2014.103. 24569470 10.1038/bjc.2014.103 PMC4007220

[26] Nordenskjöld AC, Bujila R, Aspelin P, Flodmark O, Kaijser M. Risk of meningioma after CT of the head. Radiology. 2017;285(2):568–75. 10.1148/radiol.2017161433. 28809584 10.1148/radiol.2017161433

[27] Burch JD, Craib KJ, Choi BC, Miller AB, Risch HA, Howe GR. An exploratory case-control study of brain tumors in adults. J Natl Cancer Inst. 1987;78(4):601–9. PMID: 3104645. 3104645

[28] Nishi M, Miyake H. A case-control study of non-T cell acute lymphoblastic leukaemia of children in Hokkaido, Japan. J Epidemiol Community Health. 1989;43(4):352–5. 10.1136/jech.43.4.352 . PMID: 2614325; PMCID: PMC1052873. 2614325 10.1136/jech.43.4.352 PMC1052873

[29] Wingren G, Hatschek T, Axelson O. Determinants of papillary cancer of the thyroid. Am J Epidemiol. 1993;138(7):482 – 91. 10.1093/oxfordjournals.aje.a116882. PMID: 8213752. 10.1093/oxfordjournals.aje.a116882 8213752

[30] Hallquist A, Hardell L, Degerman A, Boquist L. Occupational exposures and thyroid cancer: results of a case-control study. Eur J Cancer Prev. 1993;2(4):345-9. 10.1097/00008469-199307000-00009. PMID: 8358287. 10.1097/00008469-199307000-00009 8358287

[31] Schildt EB, Eriksson M, Hardell L, Magnuson A. Oral infections and dental factors in relation to oral cancer: a Swedish case–control study. Eur J Cancer Prev. 1998;7(3):201-6. 10.1097/00008469-199806000-00004. PMID: 9696928. 10.1097/00008469-199806000-00004 9696928

[32] Rodvall Y, Ahlbom A, Pershagen G, Nylander M, Spännare B. Dental radiography after age 25 years, amalgam fillings and tumours of the central nervous system. Oral Oncol. 1998;34(4):265–9. PMID: 9813721. 9813721

[33] Ma H, Hill CK, Bernstein L, Ursin G. Low-dose medical radiation exposure and breast cancer risk in women under age 50 years overall and by estrogen and progesterone receptor status: results from a case-control and a case-case comparison. Breast Cancer Res Treat. 2008;109(1):77–90. 10.1007/s10549-007-9625-5. Epub 2007 Jul 7. PMID: 17616809. 10.1007/s10549-007-9625-5 17616809

[34] Memon A, Godward S, Williams D, Siddique I, Al-Saleh K. Dental x-rays and the risk of thyroid cancer: a case-control study. Acta Oncol. 2010;49(4):447–53. 10.3109/02841861003705778. 20397774 10.3109/02841861003705778

[35] Davis F, Il’yasova D, Rankin K, McCarthy B, Bigner DD. Medical diagnostic radiation exposures and risk of gliomas. Radiat Res. 2011;175:790–6. 10.1667/RR2186.1. 21466382 10.1667/RR2186.1 PMC3151669

[36] Parodi S, Santi I, Marani E, Casella C, Puppo A, Sola S, Fontana V, Stagnaro E. Chronic diseases, medical history and familial cancer, and risk of leukemia and non-Hodgkin’s lymphoma in an adult population: a case-control study. Cancer Causes Control. 2015;26(7):993–1002. 10.1007/s10552-015-0592-6. 25982034 10.1007/s10552-015-0592-6

[37] Zhang Y, Chen Y, Huang H, Sandler J, Dai M, Ma S, Udelsman R. Diagnostic radiography exposure increases the risk for thyroid microcarcinoma: a population-based case-control study. Eur J Cancer Prev. 2015;24(5):439–46. 10.1097/CEJ.0000000000000169. 25932870 10.1097/CEJ.0000000000000169 PMC4516577

[38] World Cancer Research Fund International. The grading criteria with the global Cancer Update (CUP Global). Version date: 10 November 2023. Available from: https://www.wcrf.org/wp-content/uploads/2024/11/CUP-Global-Grading-Criteria_November-2023.pdf.

[39] Memon A, Rogers I, Paudyal P, Sundin J. Dental X-rays and the risk of thyroid cancer and meningioma: a systematic review and meta-analysis of current epidemiological evidence. Thyroid. 2019;29(11):1572–93. 10.1089/thy.2019.0105. 31502516 10.1089/thy.2019.0105

[40] Chang LA, Miller DL, Lee C, Melo DR, Villoing D, Drozdovitch V, Thierry-Chef I, Winters SJ, Labrake M, Myers CF, Lim H, Kitahara CM, Linet MS, Simon SL. Thyroid radiation dose to patients from diagnostic radiology procedures over eight decades: 1930–2010. Health Phys. 2017;113(6):458–73. 10.1097/HP.0000000000000732. 28968349 10.1097/HP.0000000000000723 PMC5677542

[41] Harrison JD, Balonov M, Bochud F, Martin CJ, Menzel HG, Smith-Bindman R, Ortiz-López P, Simmonds JR, Wakeford R. The use of dose quantities in radiological protection: ICRP publication 147 Ann ICRP 50(1) 2021. J Radiol Prot. 2021;41(2). 10.1088/1361-6498/abe548. PMID: 33571972. 10.1088/1361-6498/abe548 33571972

[42] Foucart JM, Felizardo R, Pizelle C. La radioprotection en orthodontie: données utiles [Radiation Protection in Orthodontics: relevant data]. Orthod Fr. 2012;83(1):3–10. 10.1051/orthodfr/2011147. 22455646 10.1051/orthodfr/2011147

[43] Xu P, Luo H, Huang GL, Yin XH, Luo SY, Song JK. Exposure to ionizing radiation during dental X-rays is not associated with risk of developing meningioma: a meta-analysis based on seven case-control studies. PLoS ONE. 2015;10(2):e0113210. 10.1371/journal.pone.0113210. 25658814 10.1371/journal.pone.0113210 PMC4319947

[44] Journy N, Laurier D, Bernier MO. Comment on: Are the studies on cancer risk from CT scans biased by indication? Elements of answer from a large-scale cohort study in France. Br J Cancer. 2015;112:1843–4. PMID: 26010503; PMCID: PMC4647234. 26010503 10.1038/bjc.2015.105 PMC4647234

[45] Little MP, Wakeford R, Borrego D, et al. Leukaemia and myeloid malignancy among people exposed to low doses (< 100 mSv) of ionising radiation during childhood: a pooled analysis of nine historical cohort studies. Lancet Haematol. 2018;5(8):e346–58. 10.1016/S2352-3026(18)30092-9. 30026010 10.1016/S2352-3026(18)30092-9 PMC6130888

[46] Hinds MW, Thomas DB, O’Reilly HP, Asbestos. dental X-rays, tobacco, and alcohol in the epidemiology of laryngeal cancer. Cancer. 1979;44(3):1114-20. 10.1002/1097-0142(197909)44:3<1114::aid-cncr2820440346>3.0.co;2-e. PMID: 476588. 10.1002/1097-0142(197909)44:3<1114::aid-cncr2820440346>3.0.co;2-e 476588

[47] Preston-Martin S, Thomas DC, White SC, Cohen D. Prior exposure to medical and dental x-rays related to tumors of the parotid gland. J Natl Cancer Inst. 1988;80(12):943-9. 10.1093/jnci/80.12.943. PMID: 3398070. 10.1093/jnci/80.12.943 3398070

[48] Hwang SY, Choi ES, Kim YS, Gim BE, Ha M, Kim HY. Health effects from exposure to dental diagnostic X-ray. Environ Health Toxicol. 2018;33(4):e2018017. 10.5620/eht.e2018017. 30661338 10.5620/eht.e2018017 PMC6341170

[49] Chauhan V, Wilkins RC. A comprehensive review of the literature on the biological effects from dental X-ray exposures. Int J Radiat Biol. 2019;95(2):107–19. 10.1080/09553002.2019.1547436. 30496029 10.1080/09553002.2019.1547436

[50] Harrison JD, McCready-Shea S, Hill MA, Smith GM, Sutton DG. Low doses of ionising radiation: definitions and contexts. J Radiol Prot. 2024 Oct 7. 10.1088/1361-6498/ad83dd. Epub ahead of print. PMID: 39374619. 10.1088/1361-6498/ad83dd 39374619

[51] Lewis SJ, Gardner M, Higgins J, et al. Developing the WCRF International/University of Bristol methodology for identifying and carrying out systematic reviews of mechanisms of exposure-cancer associations. Cancer Epidemiol Biomarkers Prev. 2017;26(11):1667–75. 10.1158/1055-9965.EPI-17-0232. Epub 2017 Oct 4. PMID: 28978562; PMCID: PMC6029666. 28978562 10.1158/1055-9965.EPI-17-0232 PMC6029666

[52] Jha N, Kim YJ, Lee Y, et al. Projected lifetime cancer risk from cone-beam computed tomography for orthodontic treatment. Korean J Orthod. 2021;51(3):189–98. 10.4041/kjod.2021.51.3.189. 33984226 10.4041/kjod.2021.51.3.189 PMC8133899

[53] Yeh JK, Chen CH. Estimated radiation risk of cancer from dental cone-beam computed tomography imaging in orthodontics patients. BMC Oral Health. 2018;18(1):131. 10.1186/s12903-018-0592-5 . PMID: 30075771; PMCID: PMC6091080. 30075771 10.1186/s12903-018-0592-5 PMC6091080

[54] Oenning AC, Jacobs R, Salmon B, DIMITRA Research Group. ALADAIP, beyond ALARA and towards personalized optimization for paediatric cone-beam CT. Int J Paediatr Dent. 2021;31(5):676–678. ( http://www.dimitra.be ). 10.1111/ipd.12797. PMID: 33844356. 10.1111/ipd.12797 33844356

[55] Van Acker JWG, Pauwels NS, Cauwels RGEC, Rajasekharan S. Outcomes of different radioprotective precautions in children undergoing dental radiography: a systematic review. Eur Arch Paediatr Dent. 2020;21(4):463–508. 10.1007/s40368-020-00544-8. 32557182 10.1007/s40368-020-00544-8

