# Supplementary figures and images for: Cancer risk from low-dose ionizing radiation in dental imaging: A systematic review and meta-analysis
Source: BMC Oral Health. 2026 May 4;26:1169. doi: 10.1186/s12903-026-08522-0 (PMC13325578; doi:10.1186/s12903-026-08522-0)

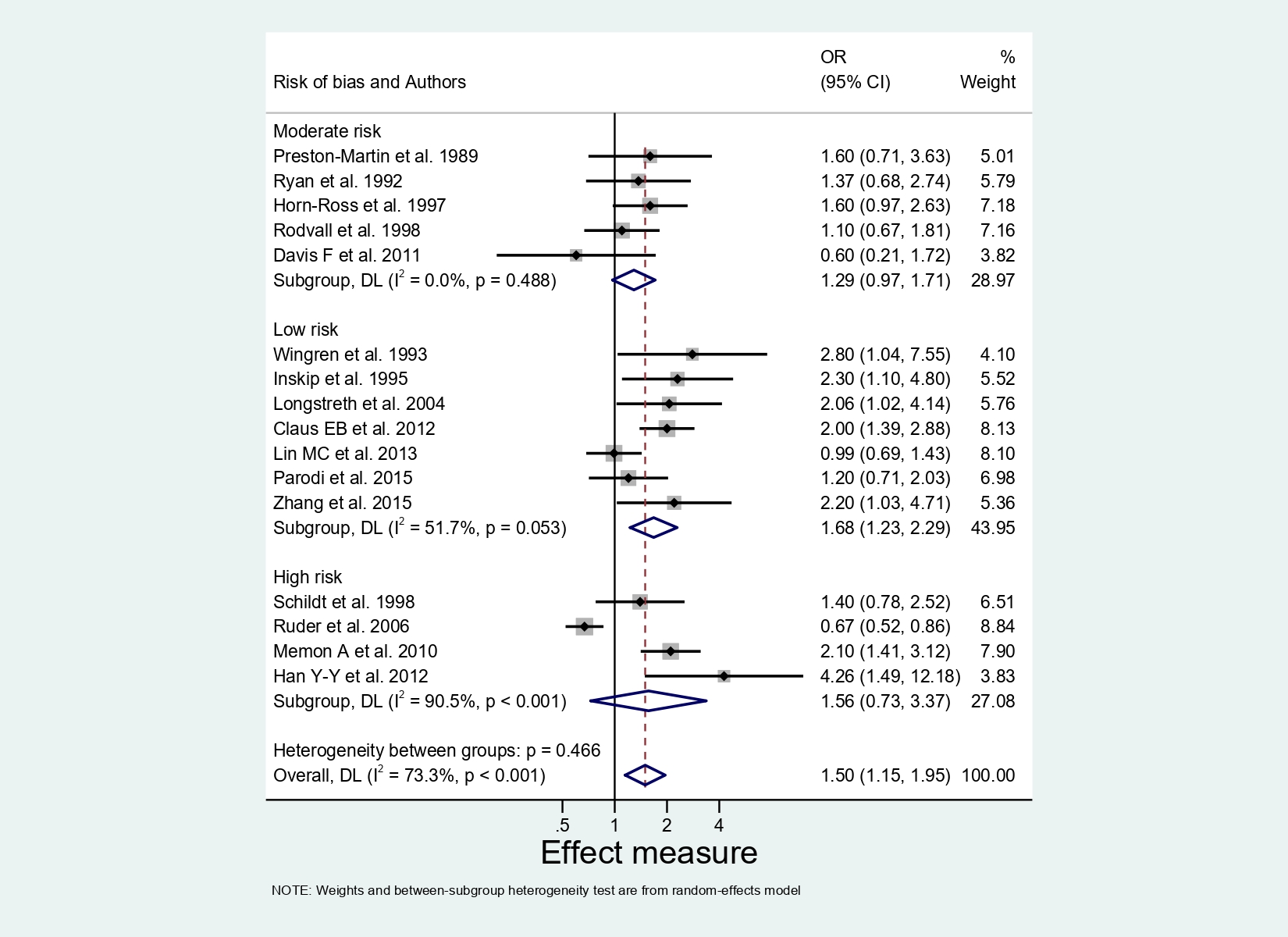

Supplement: Supplementary file 1 — Supplementary Material 1: Figure 1: Forest plot of cancer risk stratified by risk of bias in case-control studies. [file 12903_2026_8522_MOESM1_ESM.jpg]

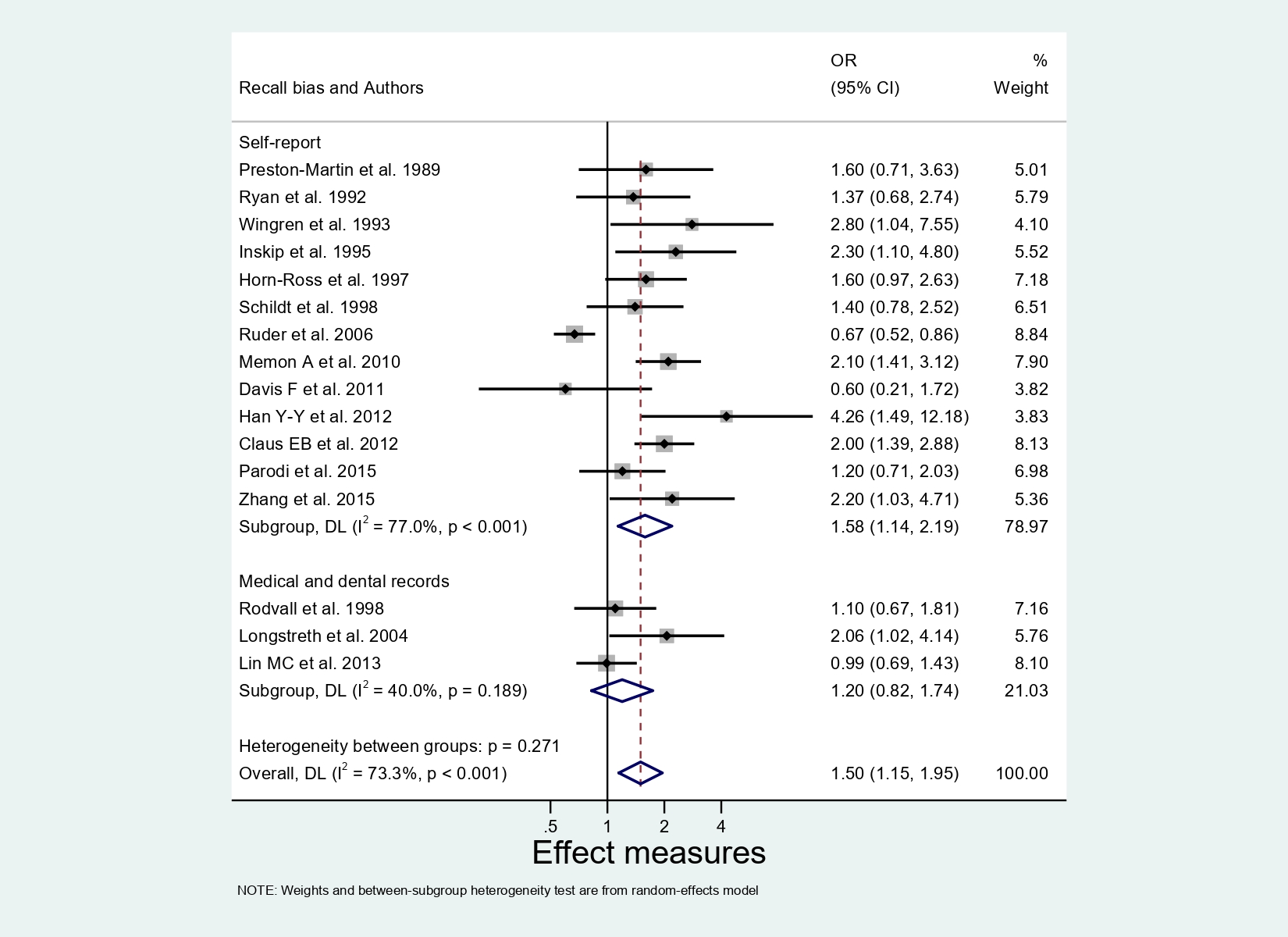

Supplement: Supplementary file 2 — Supplementary Material 2: Figure 2: Forest plot of cancer risk stratified by exposure ascertainment method. [file 12903_2026_8522_MOESM2_ESM.jpg]

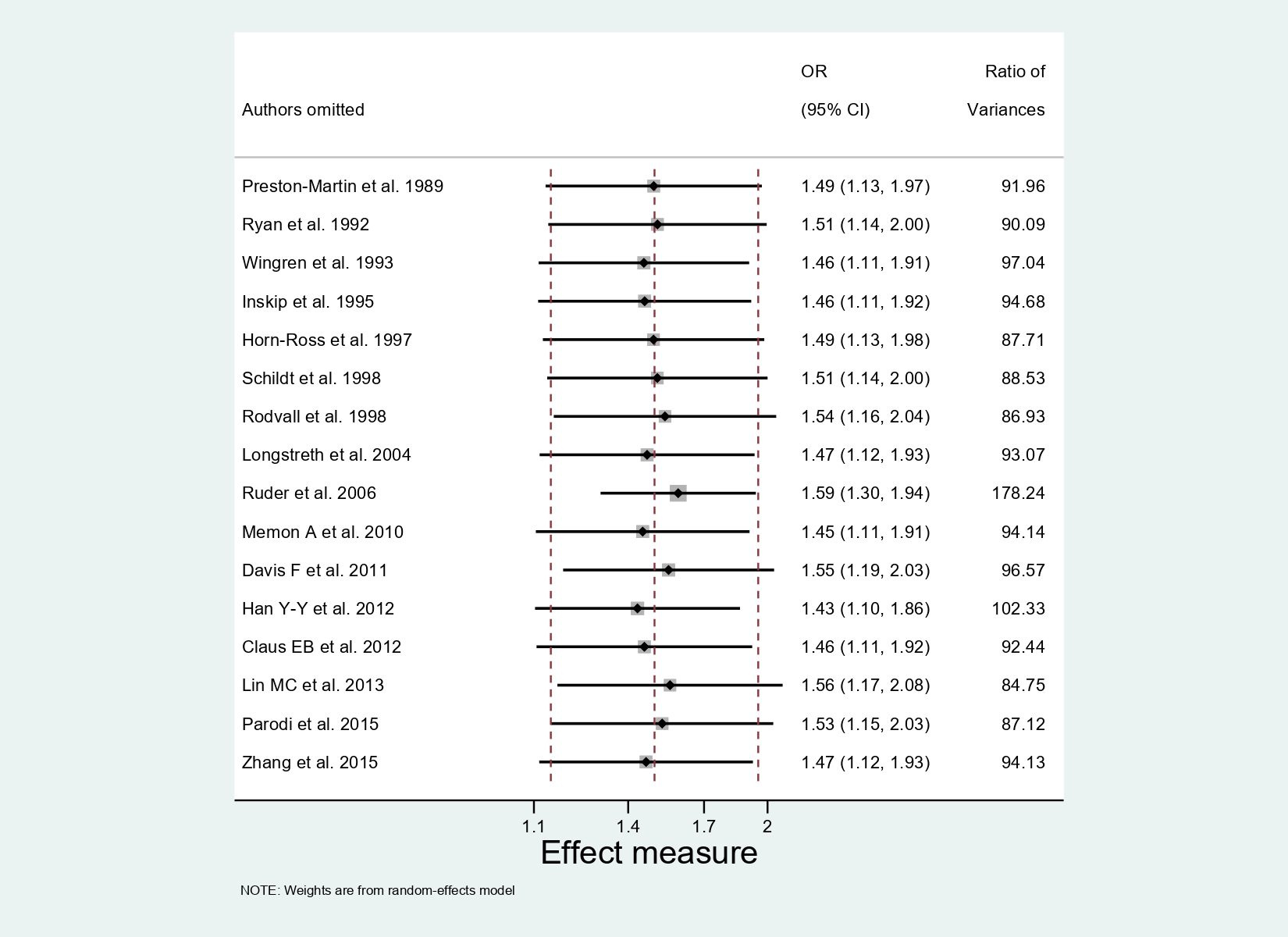

Supplement: Supplementary file 3 — Supplementary Material 3: Figure 3: Leave-one-out sensitivity analysis for case-control studies. [file 12903_2026_8522_MOESM3_ESM.jpg]

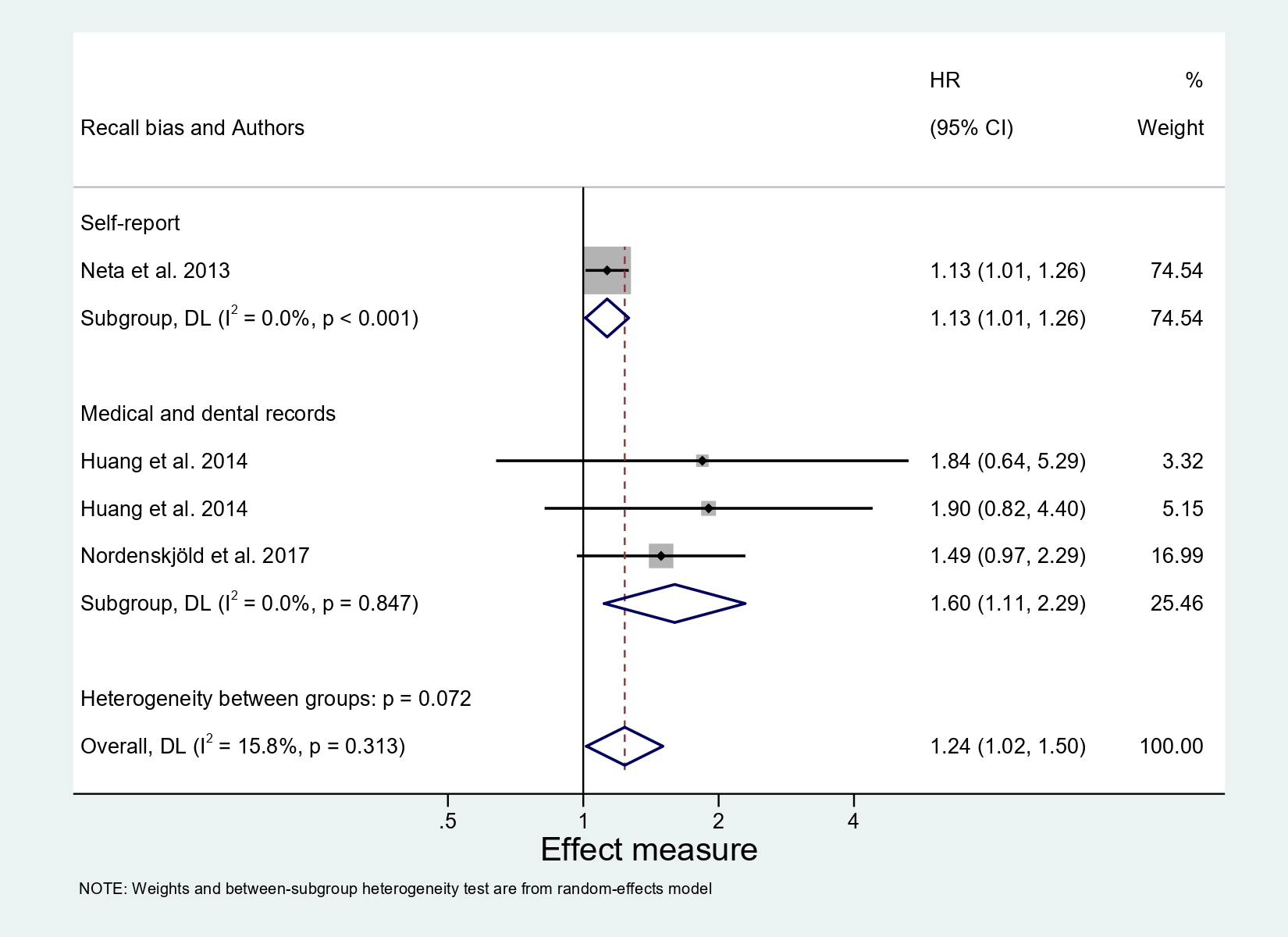

Supplement: Supplementary file 4 — Supplementary Material 4: Figure 4: Forest plot of cancer risk stratified by exposure ascertainment method cohort studies. [file 12903_2026_8522_MOESM4_ESM.jpg]

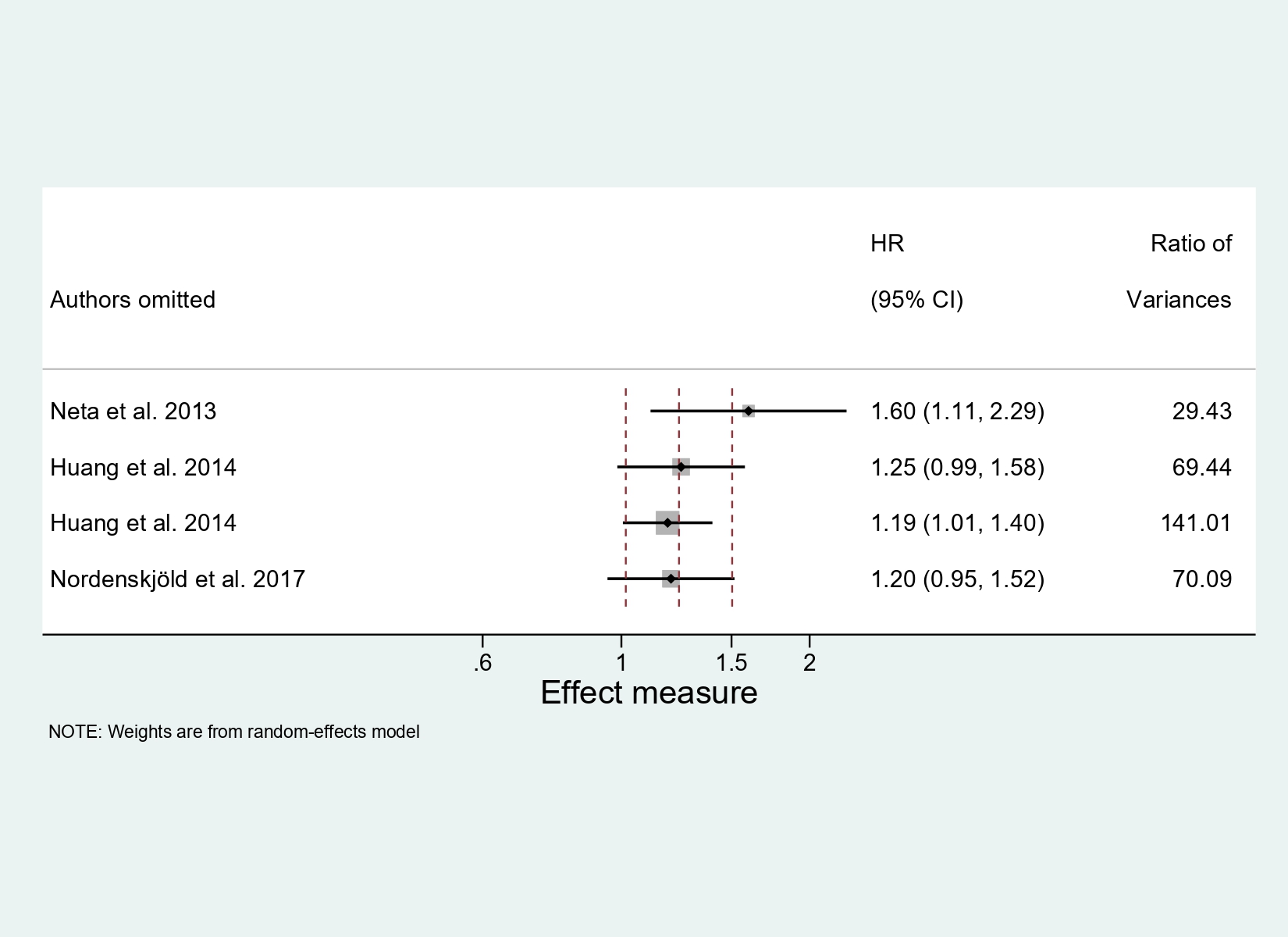

Supplement: Supplementary file 5 — Supplementary Material 5: Figure 5: Leave-one-out sensitivity analysis for cohort studies. [file 12903_2026_8522_MOESM5_ESM.jpg]

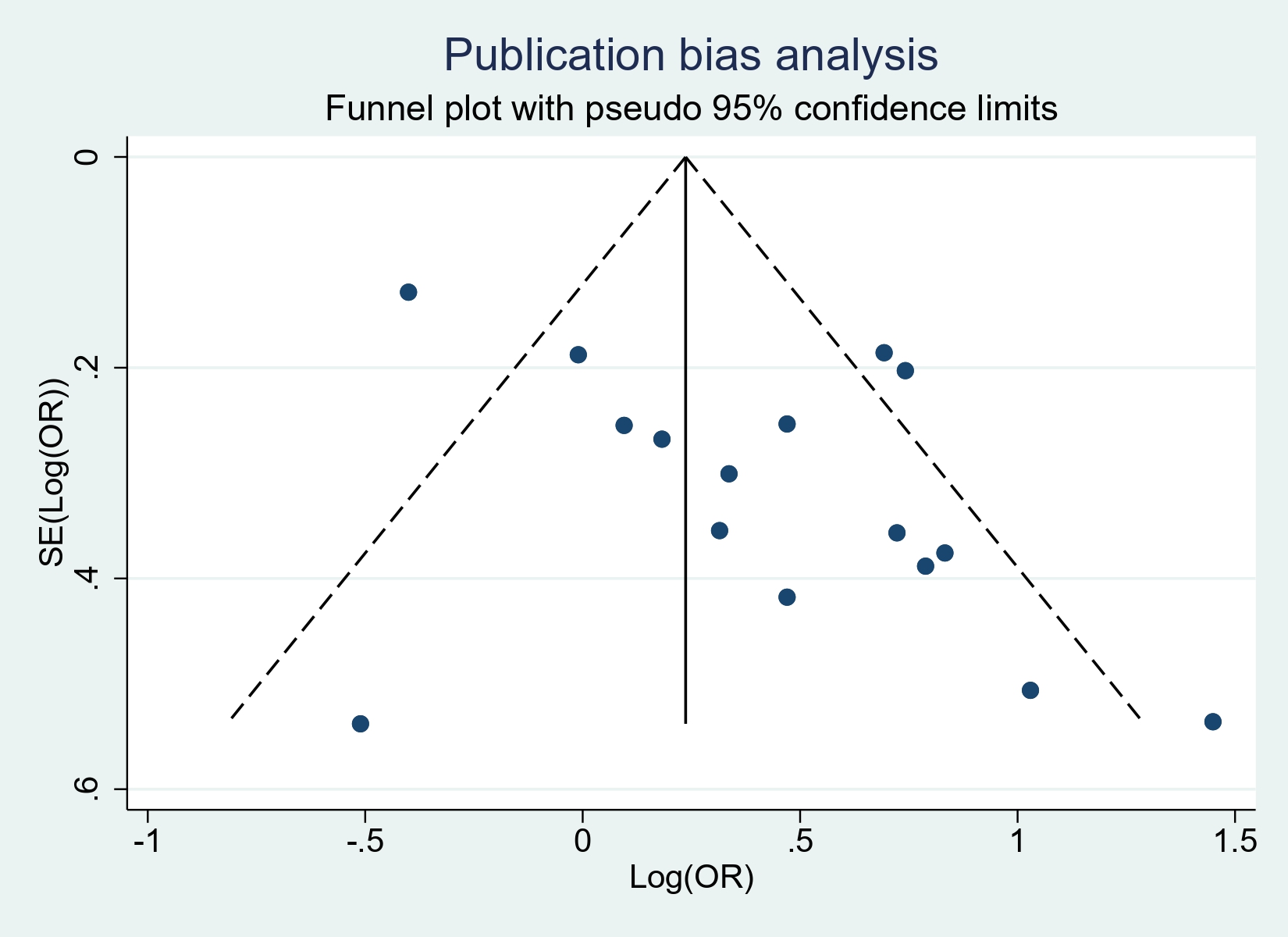

Supplement: Supplementary file 6 — Supplementary Material 6: Figure 6: Funnel plot for publication bias assessment in case-control studies. [file 12903_2026_8522_MOESM6_ESM.jpg]
